# An injectable photo-cross-linking silk hydrogel system augments diabetic wound healing in orthopaedic surgery through spatiotemporal immunomodulation

**DOI:** 10.1186/s12951-022-01414-9

**Published:** 2022-05-14

**Authors:** Jiawei Mei, Jun Zhou, Lingtong Kong, Yong Dai, Xianzuo Zhang, Wenqi Song, Chen Zhu

**Affiliations:** 1 Department of Orthopaedics, First Affiliated Hospital of University of Science and Technology of China, Hefei, 230001 China; 2 Department of Orthopaedics, Shanghai Jiao Tong University Affiliated Sixth People’s Hospital, Shanghai Jiao Tong University, Shanghai, 200233 China; 3 Department of Ophthalmology, Shanghai Jiao Tong University Affiliated Sixth People’s Hospital, Shanghai Jiao Tong University, Shanghai, 200233 China

**Keywords:** Spatiotemporal immunomodulation, Metformin, Orthopaedic wound, Macrophage polarisation, Neutrophil extracellular traps

## Abstract

**Background:**

The complicated hyperglycaemic and chronic inflammation of diabetic wounds in orthopaedic surgery leads to dysregulated immune cell function and potential infection risk. Immune interventions in diabetic wounds face a possible contradiction between simultaneous establishment of the pro-inflammatory microenvironment in response to potential bacterial invasion and the anti-inflammatory microenvironment required for tissue repair. To study this contradiction and accelerate diabetic-wound healing, we developed a photocurable methacryloxylated silk fibroin hydrogel (Sil-MA) system, co-encapsulated with metformin-loaded mesoporous silica microspheres (MET@MSNs) and silver nanoparticles (Ag NPs).

**Results:**

The hydrogel system (M@M–Ag–Sil-MA) enhanced diabetic-wound healing via spatiotemporal immunomodulation. Sil-MA imparts a hydrogel system with rapid in situ Ultra-Violet-photocurable capability and allows preliminary controlled release of Ag NPs, which can inhibit bacterial aggregation and create a stable, sterile microenvironment. The results confirmed the involvement of Met in the immunomodulatory effects following spatiotemporal dual-controlled release via the mesoporous silica and Sil-MA. Hysteresis-released from Met shifts the M1 phenotype of macrophages in regions of diabetic trauma to an anti-inflammatory M2 phenotype. Simultaneously, the M@M–Ag–Sil-MA system inhibited the formation of neutrophil extracellular traps (NETs) and decreased the release of neutrophil elastase, myeloperoxidase, and NETs-induced pro-inflammatory factors. As a result of modulating the immune microenvironmental, the M@M–Ag–Sil-MA system promoted fibroblast migration and endothelial cell angiogenesis in vivo, with verification of enhanced diabetic-wound healing accompanied with the spatiotemporal immunoregulation of macrophages and NETs in a diabetic mouse model.

**Conclusions:**

Our findings demonstrated that the M@M–Ag–Sil-MA hydrogel system resolved the immune contradiction in diabetic wounds through spatiotemporal immunomodulation of macrophages and NETs, suggesting its potential as a promising engineered nano-dressing for the treatment of diabetic wounds in orthopaedic surgery.

**Graphical Abstract:**

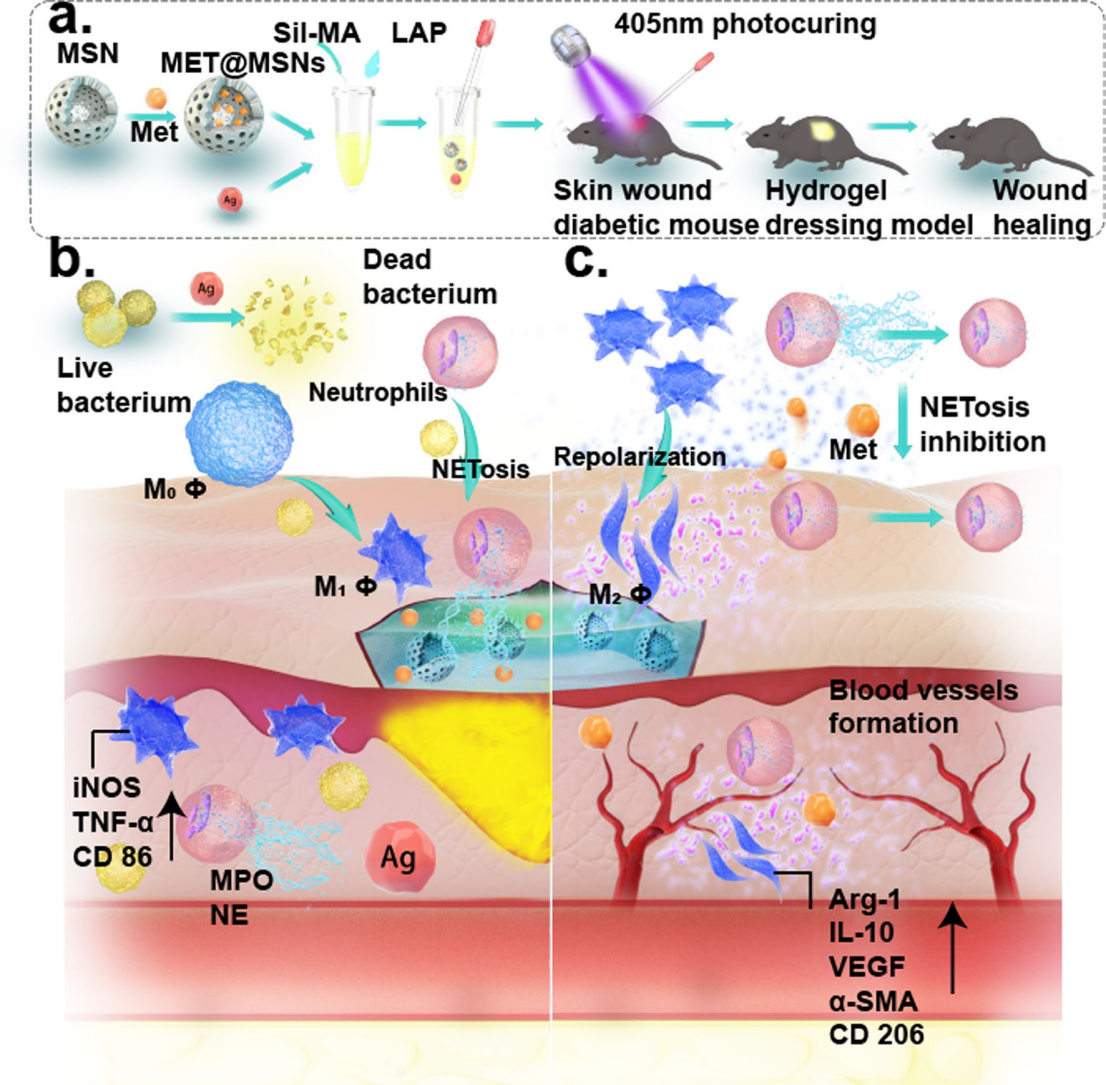

**Supplementary Information:**

The online version contains supplementary material available at 10.1186/s12951-022-01414-9.

## Background

The normal wound-healing process is a complicated series of cellular and biochemical cascade reactions through four ordered sequential but overlapping phases: haemostasis, inflammation, proliferation, and remodelling [1]. However, diabetic wounds fail to heal successfully due to the high-glucose pathological environment, which triggers local immune dysfunction, microangiopathy, and impaired tissue repair, resulting in chronic inflammatory wounds [2, 3]. Moreover, diabetic wounds are also at a higher risk of bacterial infection because of skin-barrier disruption, a sustained hyperglycaemic environment, and dysregulated immune cell function [4, 5]. Implant-associated infections due to non-healing diabetic wounds after orthopaedic surgery are devastating and can lead to immediate implant failure, revision surgery, or even amputation due to infection [6–8]. Therefore, manipulation of the complicated inflammatory microenvironment of diabetic wounds may be the most important approach for treating this clinical challenge [9]. However, interventions in diabetic wounds are complicated due to the simultaneous necessity to maintain a pro-inflammatory microenvironment capable of controlling bacterial infection and an anti-inflammatory microenvironment required for much needed tissue repair [10]. Specifically, a balance in these microenvironments is complicated by the presence of large amounts of reactive oxygen species (ROS), inflammatory factors, and inflammatory chemokines in diabetic wounds [11, 12]. ROS clearance and anti-inflammatory immune modulation facilitate wound healing but are detrimental to defence against potential infection [13, 14]. This suggests that exclusive promotion of anti-inflammatory intervention can indirectly promote potential bacterial invasion, especially in diabetic wounds [15, 16]. Thus, reconciling the ‘immune contradiction’ in diabetic wounds may be the most important issue to be addressed.

Metformin (Met) is a classical oral hypoglycaemic agent used for the treatment of type 2 diabetes. Previous studies have shown that Met has good immunomodulatory properties in vivo and in vitro and an especially significant anti-inflammatory capacity in a lipopolysaccharide (LPS)-induced inflammatory environment via Notch1 signalling or AMP-activated protein kinase activation [17]. Exogenous Met can induce macrophages to polarise toward the anti-inflammatory phenotype M2, resulting in the secretion of a series of anti-inflammatory cytokines, such as vascular endothelial growth factor (VEGF) and transforming growth factor-β, to promote tissue repair [18, 19]. The silica-based materials have been deemed safe by the United States Food and Drug Administration for medicinal applications [20]. Mesoporous silicon nanoparticles (MSNs) are porous microspheres with internal mesoporous structures that facilitate the storage and controlled release of drugs [21, 22]. MSNs can be used for drug storage due to their large surface area and pore volume, appropriate pore size, low density, thermal insulation, and permeability. These advantages have made them applicable in various biomedical applications, including tumour treatment [22], drug delivery platform [23], anti-infective [24], biocatalysts [25]. The use of MSN-loaded metformin (MET@MSNs) is an effective drug-delivery method for the controlled release and topical application, with previous studies demonstrating that MET@MSNs exhibit a well-controlled release capacity and immunomodulatory properties for the treatment of tumours [22, 26].

Silk fibroin hydrogel is an injectable hydrogel with good biocompatibility and has been used as a biological scaffold in various cells and tissues [27]. Additionally, silk fibroin hydrogel features a multiporous structure and controlled drug-delivery capability [28]. Following rapid in situ photocuring, methacryloxylated silk fibroin hydrogels (Sil-MA) exhibit extremely high adhesion and sealing properties, making them ideal wound dressings [29, 30]. Silver nanoparticles (Ag NPs) exhibit a high degree of antibacterial activity against multiple Gram-positive and Gram-negative bacteria and essential for unknown pathogenic microorganisms in wounds. Therefore, these NPs have been widely used in medical devices and antimicrobial excipients. Additionally, Ag NPs can overcome drug resistance because of their specific mechanisms of antibacterial activity, including initiation of membrane damage, interference with DNA/RNA replication, and generation of oxidative stress [31]. Moreover, Ag NPs can provide durable broad-spectrum bacterial inhibition in wounds relative to conventional antibiotics that require additional testing of drug susceptibility to determine drug class. Previous studies have demonstrated the effectiveness of silver nanoparticles-loaded silk fibroin hydrogels (Ag–Sil-MA) in accelerating wound healing through their antibacterial and promotional collagen-deposition abilities [32, 33]. Thus, Ag–Sil-MA represents an optimal antibacterial-dressing vehicle with the potential to further load immunomodulatory drugs for the treatment of complicated inflammatory diabetic wounds.

**Scheme 1:**
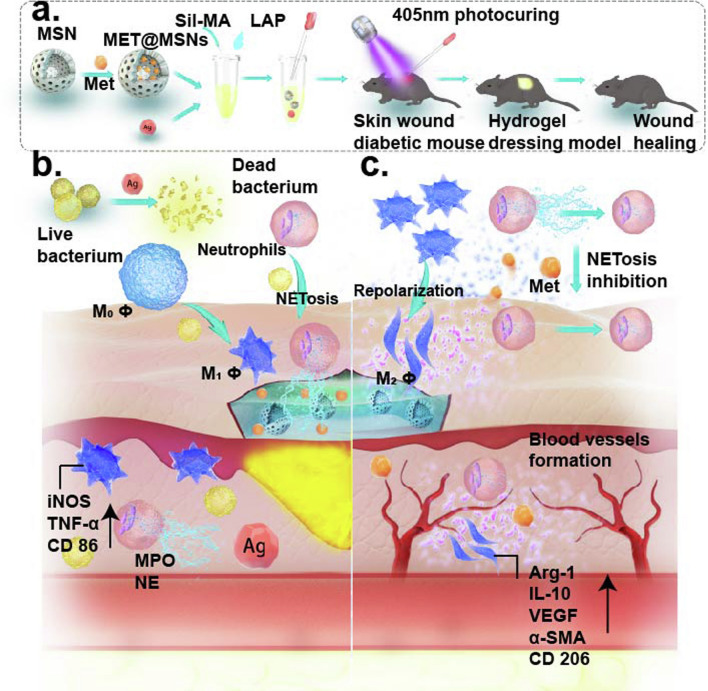
**a** Synthesis of MET@MSNs and Ag NPs-loaded Sil-MA hydrogel (M@M–Ag–Sil-MA). **b** Preliminary controlled release of Ag NPs inhibits bacterial aggregation and creates a sterile microenvironment. **c** Dual-controlled release of Met promotes wound healing through spatiotemporal immunomodulation of macrophages and NETs

In this study, we employed Ag NPs-loaded Sil-MA as an antibacterialdressing vehicle which was further loaded with MET@MSNs as an immunomodulatory drug (M@M–Ag–Sil-MA) (Scheme 1a). After in situ photocuring in diabetic wounds, bacterial inhibition is initiated by the primary release of Ag NPs from the M@M–Ag–Sil-MA hydrogel system, with macrophage immunomodulation initiated by the dual spatiotemporal controlled release of MET to promote M2 polarisation, thereby leading to tissue repair and reconstruction (Scheme 1b, c). The results demonstrated a method for spatiotemporal immunomodulation in diabetic wounds using different systems to retard drug release and with the prospect of further translation and clinical trials.

## Results and discussion

### Characterisation of MET@MSNs and M@M–Ag–Sil-MA

**Fig. 1:**
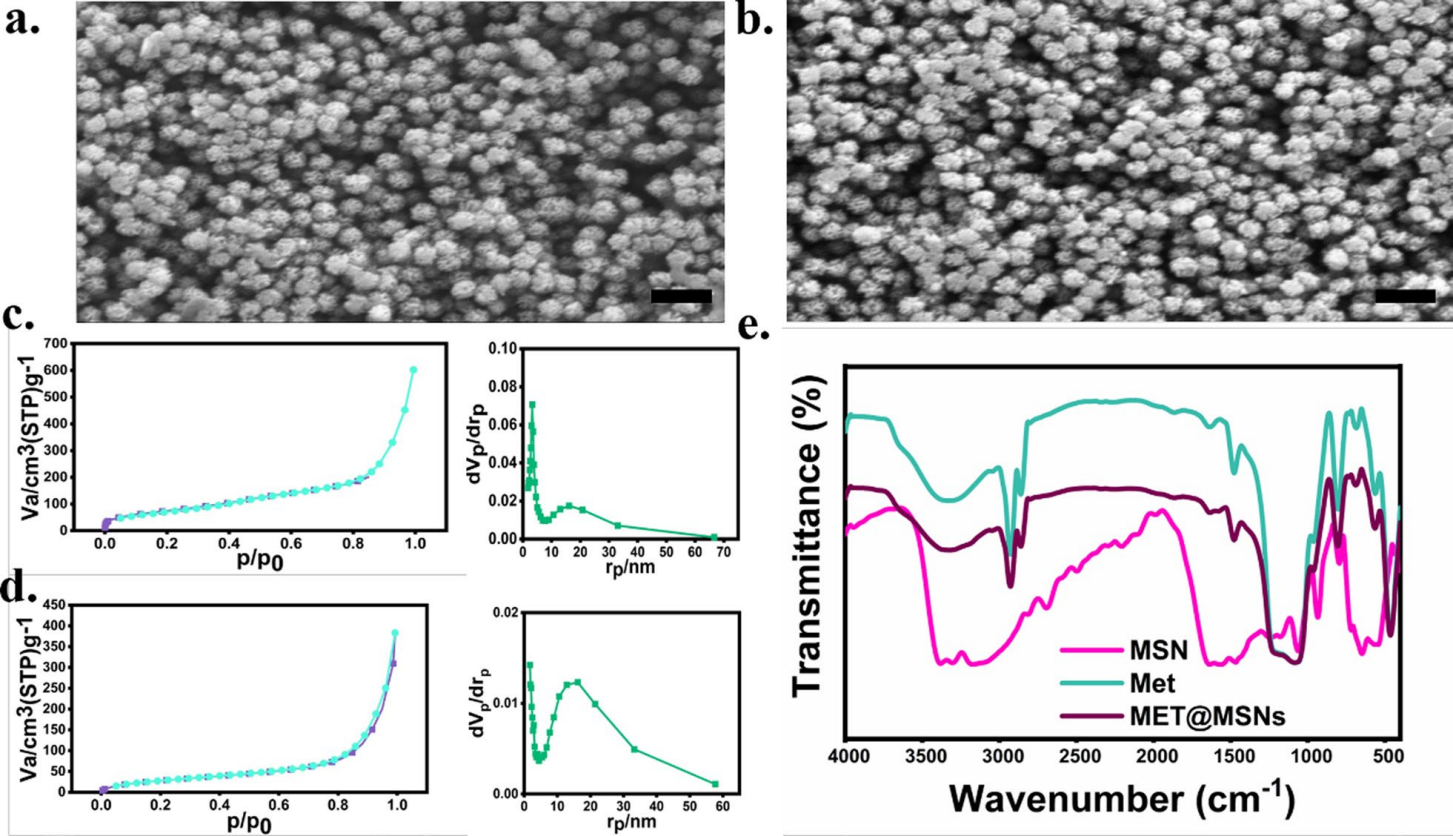
Characterization of the synthesized nanoparticles. SEM micrographs showed the average particle size of **a** MSN and **b** MET@MSNs (Scale bar: 200 μm). BET and BJH indicated the surface area and pore diameter of **c** MSN and **d** MET@MSNs. **e** FT-IR spectra of MSN, Met, and MET@MSNs

After synthesizing porous MET@MSNs nanospheres according to a previously described method [26], we evaluated the morphological characteristics of MSN and MET@MSNs using scanning electron microscopy (SEM) (Fig. 1a, b). Our results indicated that the silica NPs showed a regular spherical morphology with good dispersion and homogeneity and an average particle size of ~ 85.81 nm. Notably, we detected significant mesopores at the surface of the drug-free particles. However, MET@MSNs exhibited a larger size (average diameter: ~ 90.71 nm) and no significant mesopores on the surface. This result was similar with that of previous studies [22, 26]. Furthermore, we determined the specific surface areas of the MSN and MET@MSNs using an N2 adsorption analyser, with the pore-size distribution and surface area analysed by Barrett–Joyner–Halenda and Brunauer–Emmett–Teller (BET) procedures, respectively (Fig. 1c, d). The results showed that the BET-specific surface area of the unloaded monodisperse silica microspheres was 307.20 m^2^/g (pore diameter, 8.73 nm), whereas the specific surface area and pore diameter of the MET@MSNs were reduced (281.11 m^2^/g and 8.48 nm, respectively). Fourier-transform infrared spectroscopy (FT-IR) results of monodisperse silica microspheres (Fig. 1e) revealed the main absorption bands at 2695 cm^−1^, 2217 cm^−1^, and 1475 cm^−1^ belonging to the C–H-stretching vibration and C–H deformation, respectively. Moreover, we attributed the absorption peaks at 1168 cm^−1^ and 1064 cm^−1^ to the external and internal asymmetric stretching vibrations of the Si–O–Si bond. We detected major absorption bands at 1485 cm^−1^ and 1084 cm^−1^ due to MET loading, which were attributed to –C=N and C=N Schiff base stretching. The N–H-stretching vibrations at 3321 cm^−1^ and N–H-deformation vibrations at 2854 cm^−1^ showed the absorption of amino groups by dispersed monodisperse NPs.

**Fig. 2:**
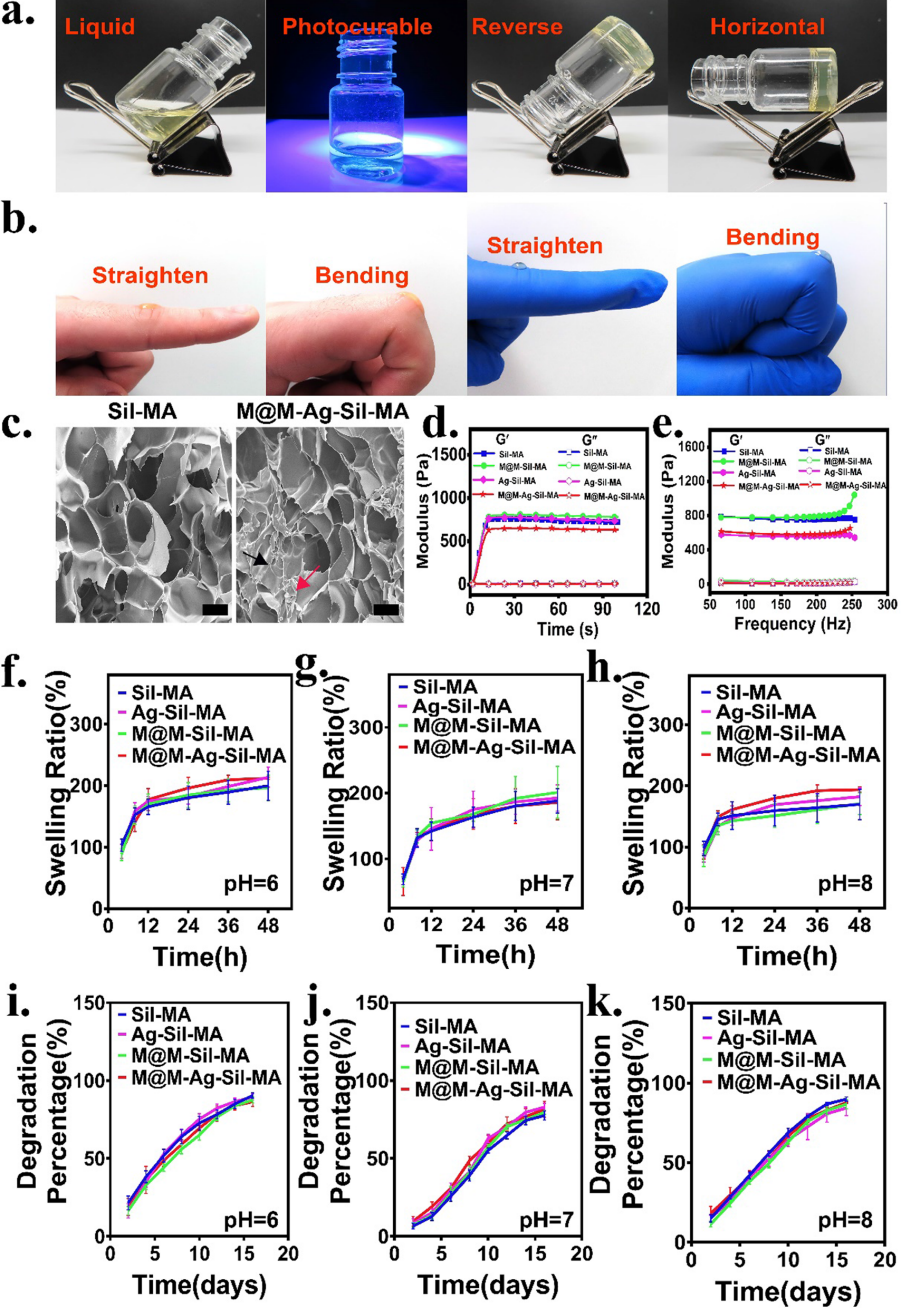
Characterization of the hydrogel system. **a** Photographs of the solution to hydrogel transition by photocuring and the stability of hydrogel was proved by placing in different orientations. **b** Adhesion and bending ability on different surfaces. **c** Representative SEM images showed the interconnected porous meshwork of Sil-MA and M@M–Ag–Sil-MA scaffolds (Scale bar: 50 μm). The black and red arrows represent MET@MSNs and Ag NPs encapsulated in hydrogels, respectively. Storage modulus (G′) surpassing the loss modulus (G″) and at different **d** time (lasting for 100 s) and **e** frequencies (from 65 to 255 Hz). **f**–**h** Swelling property of hydrogel system containing different components under the PBS with different pH values (pH = 6.0/7.4/8.0). **i**–**k** Degradation property of the different hydrogel system soaked into PBS until complete swelling under different pH values (pH = 6.0/7.4/8.0)

Ag NPs use in biomedical applications has gradually increased because of its antibacterial activity; however, the inferior surface-binding affinity of Ag NPs suggests the necessity of their use with hydrogels, which demonstrate a good cross-linking three-dimensional structure and a hydrophilic network of polymers that act as water-entrapping scaffolds and a vehicle for Ag NPs delivery [34]. A previous study reported the optimal concentration of Ag NPs colloids loaded in hydrogels for antimicrobial and wound-healing promotion [35]. In the present study, we used the same concentration ratios of Ag NPs colloid concentrations as antimicrobial agents for the hydrogel system. Different ratios of the MET@MSNs aqueous dispersion and stationary terminal concentrations of Ag NPs colloids were incorporated into the Sil-MA hydrogel-precursor solution, and photocuring was performed by UV radiation at 405 nm in the presence of a photo initiator (lithium phenyl-2,4,6-trimethylbenzoylphosphinate, LAP). Synthesis of M@M–Ag–Sil-MA (Fig. 2a) revealed a photocured hydrogel that maintained its morphology in an inverted or horizontal position and demonstrated good adhesion, deformation, and writing abilities at the finger joints (with or without nitrile gloves) and on hairy skin with or without nitrile gloves when inverted (Fig. 2b, Additional file 1: Fig. S1 and S2). SEM assessment (Fig. 2c) of the surface morphologies of the developed hydrogel system resulted in micrographs showing the interconnected porous meshwork of Sil-MA and M@M–Ag–Sil-MA scaffolds with void sizes > 100 μm, which encouraged cell adhesion, proliferation, and migration within the hydrogels [36, 37]. Additionally, rheological data confirmed the successful preparation of the M@M–Ag–Sil-MA hydrogel system, with the storage modulus (G′) surpassing the loss modulus (G″) immediately after activation by 405 nm UV radiation and lasting for 100 s and promoting the rapid in situ photo curability of the hydrogel system (Fig. 2d). However, upon the stable formation of hydrogels, G′ was independent of the shear frequency from 65 to 255 Hz, indicating that the hydrogel system was robust and suitable for application as a wound dressing (Fig. 2e). To demonstrate the ability of this system to absorb tissue fluid, testing of the swelling effect of the hydrogel system at different pH values revealed a swelling rate of 200% (Fig. 2f–h) and an ability to maintain a limited swelling ratio at different pH values (pH = 6.0, 7.4, and 8.0), with no significant changes observed due to drug loading. Our findings suggested the potential stability of the M@M–Ag–Sil-MA hydrogel system in infected wound applications according to its compatibility with the wound area without over-swelling. Further observations indicated that the degradation of the hydrogel system continued until day 16, with the degradation time slightly faster in acidic (pH = 6.0) and alkaline (pH = 8.0) environments than in neutral (pH = 7.4) environments (Fig. 2i–k). To more realistically simulate the in vivo environment, we supplemented serum and papain groups as controls (Additional file 1: Fig. S3). The degradation time of all hydrogels exceeded 2 weeks, which is sufficient for the time required for wound healing. Despite the acidic nature of fresh diabetic wounds and the gradual shift to alkaline as the wounds persist, the hydrogel system continues to degrade steadily in the corresponding pH environment. These results suggested the suitability of the hydrogel characteristics as wound dressings for orthopaedic patients with diabetes.

### Dual-controlled drug release and biocompatibility of the M@M–Ag–Sil-MA hydrogel in vitro

**Fig. 3:**
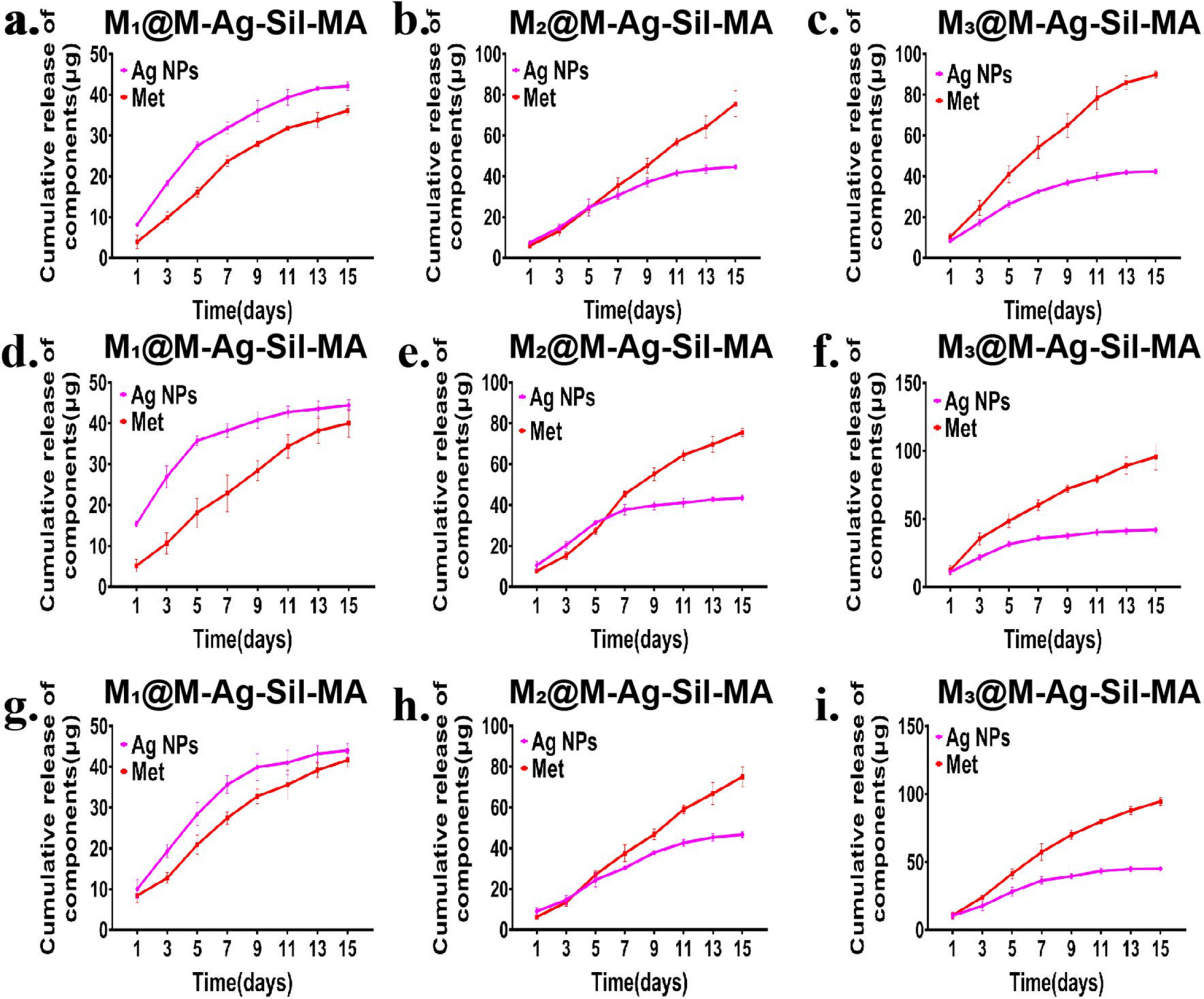
Drug release assay of the hydrogel system in vitro. Cumulative release mass curves of Met and Ag NPs of hydrogel systems with different MET@MSNs and Ag NPs mass ratios (1: 1, 2:1, and 3:1) in 2 mL neural (pH = 7.4) (**a**–**c**), acidic (pH = 6.0) (**d**–**f**) and alkaline(pH = 8.0) (**g**–**i**) PBS at 37 ℃ using a shaker (200 rpm)

We then determined the release of Ag NPs and Met from the M@M–Ag–Sil-MA hydrogel system by UV–vis spectroscopy. Specifically, hydrogel systems with different MET@MSNs and Ag NPs mass ratios (1:1, 2:1, and 3:1) were immersed in liquid environments with different pH values (pH = 6.0, 7.4, and 8.0) to observe the cumulative concentration of the components in the environment. At a mass ratio of 1:1, the cumulative release concentration of Ag NPs was consistently higher than the cumulative release concentration of Met, while at a mass ratio of 3:1, the cumulative release concentration of Met was consistently higher than the cumulative release concentration of Ag NPs. When the mass ratio was 2:1, the cumulative release concentration of Ag NPs was higher than the cumulative release concentration of Met on day 5, whereas the cumulative release concentration and percentage of Met was consistently higher than the cumulative release concentration of Ag NPs after day 7 (Fig. 3a–c and Additional file 1: Fig. S6a–c). The release of the hydrogel system was accelerated in acidic (Fig. 3d–f and Additional file 1: Fig. S6d–f) and alkaline environments (Fig. 3g–i and Additional file 1: Fig. S6g–i), whereas the release order of Ag NPs and Met were unaltered. This possibility of differential sequential release arises from the combined application of different controlled-release systems. The release of hydrogel-loaded drugs depends to a large extent on hydrogel degradation and dissolution. Although Met is a smaller drug molecule relative to Ag NPs, the peak concentration time of Met occurs after that of Ag NPs under the delay of the dual controlled-release system of mesoporous silica and Sil-MA. It is the difference in release efficiency in different spaces that causes this difference in the time of drug release. Such spatiotemporal regulation provides the possibility of spatiotemporal immunomodulation.

Furthermore, we determined the biocompatibility of the M@M–Ag–Sil-MA hydrogel system with cells. First, fluorescence microscopy observations after co-culture of EA.hy 926 cells with M@M-Sil-MA_1_ and M@M-Sil-MA_7_ harbouring different Ag NPs and MET@MSNs mass ratios showed favourable cytoskeletal morphology (Additional file 1: Fig. S7). Live/dead assays showed that RAW264.7 cells were viable on M@M–Ag–Sil-MA, with > 90% viability after co-culture with all M@M–Sil-MA_*1*_ and M@M–Sil-MA_*7*_ [conditioned medium (CM) from hydrogel culture on days 1 and 7, respectively (Additional file 1: Fig. S8). Quantification of L929 cells viability by the Cell Counting Kit-8 assay after co-culture with all M@M–Sil-MA_*1*_ and M@M–Sil-MA_*7*_ (Additional file 1: Fig. S9) indicated similar optical density values in all groups with no statistical differences. These results of the drug-release experiments and cytocompatibility assays demonstrated the controlled drug release and safety of the M@M–Ag–Sil-MA hydrogel system. To illustrate the spatiotemporal modulation of the hydrogel system, we chose hydrogels with a mass ratio of MET@MSNs to Ag NPs of 2:1 for subsequent experiments.

### Antibacterial performance of the M@M–Ag–Sil-MA hydrogel in vitro

**Fig. 4:**
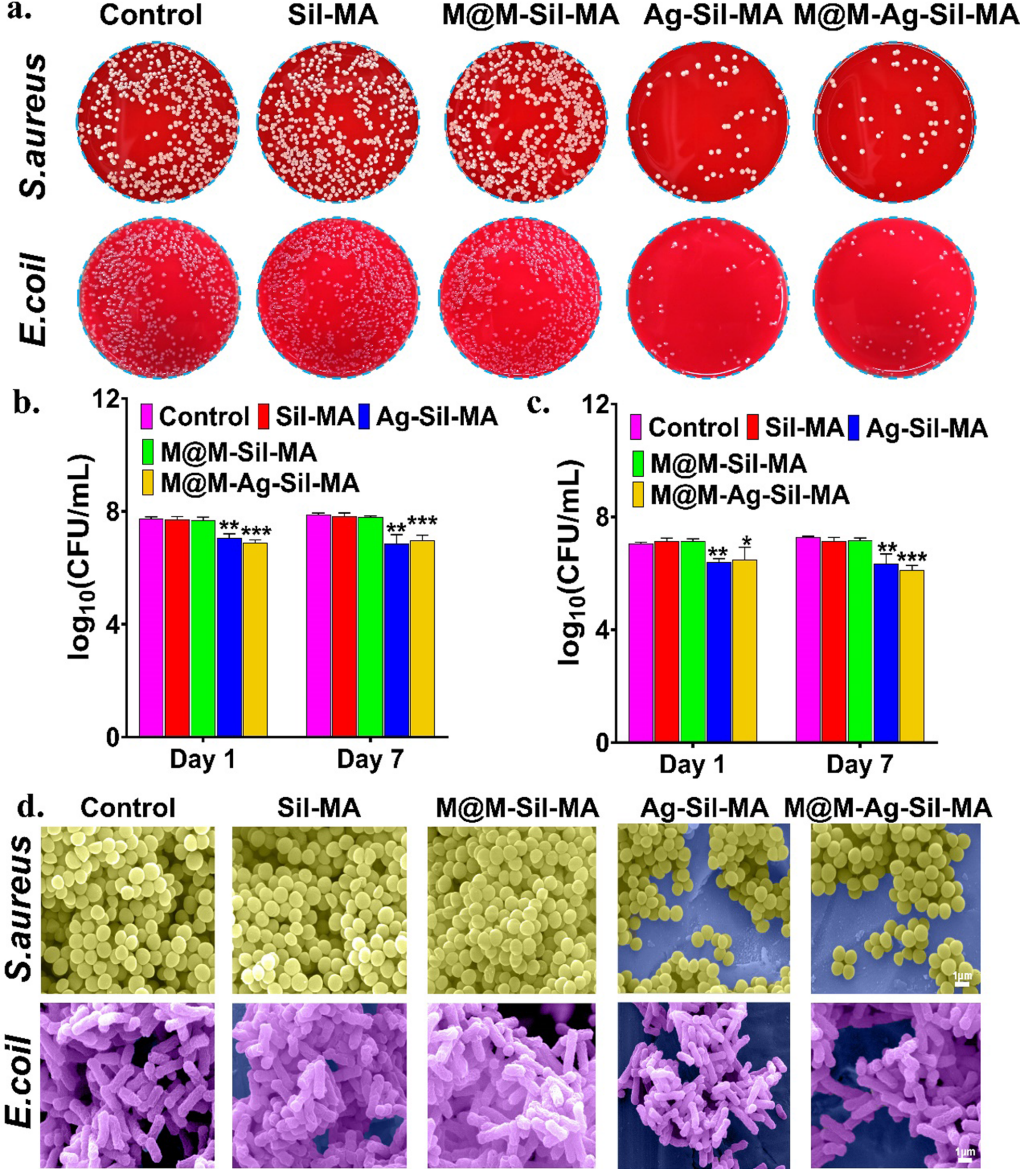
Antibacterial properties of the hydrogel systems. **a** Representative culture images of bacterial colonies formed by *S. aureus* (the top row) and *E. coli* (the bottom row) after exposure to PBS, CMs of Sil-MA, M@M-Sil-MA, Ag-Sil-MA, M@M-Ag-Sil-MA and the relative amounts of the corresponding colonies of *S. aureus* (**b**) and *E. coli* (**c**) determined by spread plate method. **d** Representative SEM images of *S. aureus* and *E. coli* treated with PBS or different CMs. Yellow spheres indicate *S. aureus*, and violet rods indicate *E. coli.* *P < 0.05, **P < 0.01 and ***P < 0.001) (Scale bar: 1 μm)

We then evaluated the ability of preliminary release of the Ag NPs incorporated into the M@M–Ag–Sil-MA hydrogel system to inhibit bacterial infection. The relative colony counts were 7.72 ± 0.10 (CFU/mL) for *Staphylococcus aureus* (*S. aureus)*and 7.15 ± 0.09 (CFU/mL) for *Escherichia coli (E. coli)*when co-cultured with CM of Sil-MA, which were decreased significantly when co-cultured with the CM of Ag–Sil-MA and M@M–Ag–Sil-MA hydrogel system, leaving only 6.90 ± 0.09 (CFU/mL) and 6.30 ± 0.43 (CFU/mL) of *S. aureus* and *E. coli*, respectively, in the M@M–Ag–Sil-MA hydrogel system group (Fig. 4a–c and Additional file 1: Fig. S10). Additionally, zone of inhibition (ZOI) tests of the hydrogel system against *S. aureus* and *E. Coli* revealed similar ZOIs for the Ag–Sil-MA and M@M–Ag–Sil-MA hydrogels to those of antibiotic-sensitive tablets but better than the Sil-MA and M@M–Sil-MA hydrogels and the blank control tablet (Additional file 1: Figs. S11–13). Despite the limited contact area of the hydrogel with bacteria, the superior porous structure and water content of the hydrogel system conferred the ability to diffuse Ag NPs. The diffusion of Ag^+^ from the Ag NPs provided the hydrogel system with sustained bacterial inhibition, a process that was accompanied by the degradation of the hydrogel system [38]. Furthermore, SEM images (Fig. 4d and Additional file 1: Fig. S14) showed that bacteria could form an intact and dense biofilm when co-cultured with the CM of Sil-MA and M@M–Sil-MA hydrogels, whereas we observed only sporadic non-biofilm-forming and ruptured bacteria after co-culture with the CM of Ag–Sil-MA hydrogel and M@M–Ag–Sil-MA hydrogel system. One reason for the difficulty in healing diabetic wounds is the persistent inflammatory cell infiltration and chronic inflammation due to the high glycaemic environment and microangiopathy in the wounds [39, 40]. The presence of bacteria and infection undoubtedly exacerbates inflammatory tropism, ultimately creating a dysfunctional immune response and promoting long-term survival of bacteria in diabetic wounds [41, 42]. Therefore, the first task in promoting trauma recovery is to expose the exogenous stimulation of the trabecular microenvironment by pathogenic bacteria, which is an essential step in reversing the chronic inflammation of the wound and transforming it into a new phase of pro-repair immunity. In summary, without first promoting antimicrobial activity, immune cells in the trabeculae will have a hard time entering the subsequent repair phase. These results confirmed that the antimicrobial activity of the M@M–Ag–Sil-MA hydrogel by releasing Ag NPs, which provided a continuous environment that promoted bacterial inhibition for wound healing [43].

### Evaluation of macrophage polarisation and function in vitro

**Fig. 5:**
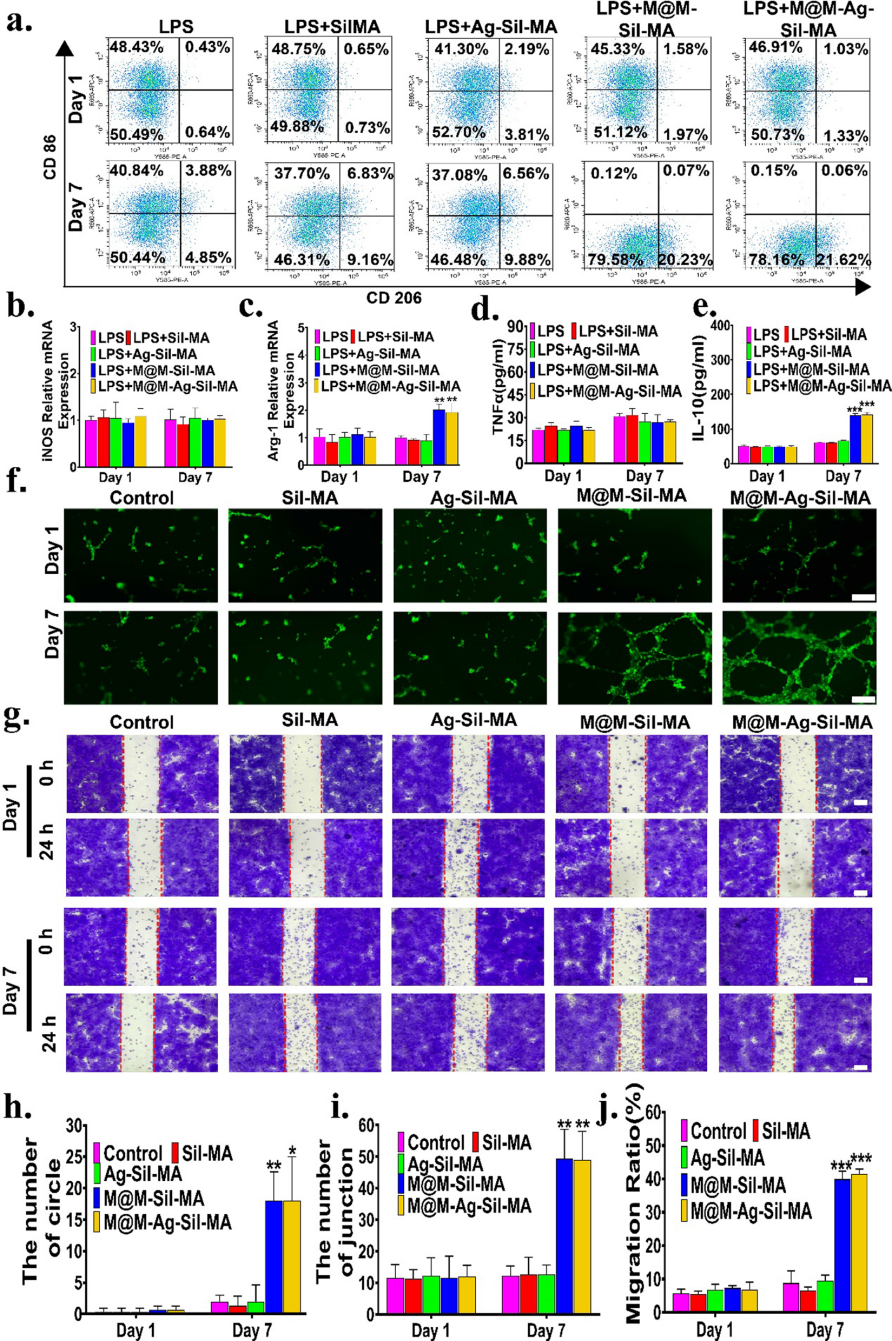
Immunomodulatory function of macrophages in vitro. **a** Representative flow cytometry results after 1 and 7 d of co-culture with CM of hydrogel systems of RAW264.7. CD 86 represents M1 macrophages surface markers and CD 206 represents M2 macrophages surface markers. **b**, **c** Expression of M1-related gene, *iNOS,* and M2-related genes,*Arg-1*after the RAW264.7 cells were treated by the day 1 and day 7 CM containing different components for 24 h. **d**, **e** ELISA results for TNF-α and IL-10 after the RAW264.7 cells were treated by different CM for 24 h. **f** In vitro angiogenesis of Ea.hy 926 cells in different MCM and comparison of circle and junction generated in 6 h of culture at 37 ℃(Scale bar: 200 μm). And the quantitative results of **h** circle and **i** junction generated were counted manually. **g** In virto migration of L929 cells were treated by the day 1 and day 7 different MCM containing different components(Scale bar: 100 μm) and **j** comparison of quantitative results of cell migration ratio in 24 h of culture at 37 ℃. *P < 0.05, **P < 0.01 and ***P < 0.001

We then evaluated the effect of the controlled release of Met following bacterial inhibition by release of Ag NPs. To mimic the in vivo inflammatory conditions of diabetes, we treated RAW264.7 cells with LPS to induce macrophage polarisation to a pro-inflammatory M1 phenotype [44, 45]. At 6-h post-induction, the medium was replaced with a new medium containing either LPS (LPS group) or CM (for days 1 and 7) of Sil-MA, M@M–Sil-MA, Ag–Sil-MA, or M@M–Ag–Sil-MA hydrogels, followed by culture for 1 day and evaluation of macrophage polarisation. Flow cytometry and reverse transcription polymerase chain reaction (RT-PCR) assay (Fig. 5a–c) results showed that all cultures with the respective CM (from 1-day cultures) showed similar results to the LPS group in terms of elevated expression of the M1 phenotype marker CD86 and inducible nitric oxide synthase (iNOS) [46, 47]. By contrast, cultures with CM from 7-days cultures (M@M–Sil-MA_*7*_ and M@M–Ag–Sil-MA_*7*_) reduced CD86 and iNOS expression but increased the expression of the M2 phenotype marker CD206 and arginase-1 (Arg-1) relative to levels observed in the LPS, Sil–MA_*7*_, and Ag–Sil-MA_*7*_ groups [48]. To detect the representative cytokines secreted by M1 and M2 macrophages after spatiotemporal immunoregulation by the M@M–Ag–Sil-MA_*7*_ hydrogel system, we measured the concentrations of tumour necrosis factor (TNF)-α and IL-10 by enzyme-linked immunosorbent assay (ELISA) (Fig. 5d, e). Cells treated with LPS and CM of M@M–Sil-MA_*1*_, Ag–Sil-MA_*1*_, and M@M–Ag–Sil-MA_*1*_ secreted similar levels of TNF-α, which is a representative pro-inflammatory factor produced by M1 macrophages. By contrast, RAW 264.7 cells treated with CM of M@M-Sil-MA_*7*_ and M@M-Ag-Sil-MA_*7*_ secreted higher levels of the anti-inflammatory cytokines IL-10, which are mainly produced by M2 macrophages. During diabetic-wound healing, the polarization of M2 macrophages contribute to limited pro-inflammatory cytokine release in the inflammatory microenvironment, as well as the release of repair cytokines for angiogenesis and cell migration, which are required for tissue repair. M@M-Ag-Sil-MA hydrogel accomplished immunomodulation of M2 polarization through a dual-controlled release of the immunomodulator Met, without affecting the level of inflammation in the pre-wound healing antimicrobial environment.

Assessment of the proangiogenic potential of the M@M–Ag–Sil-MA hydrogel via tube-formation assays (Fig. 5f) revealed that none of the endothelial cells co-incubated with day-1 medium conditioned with M2 macrophages (MCM) for 6 h on the Matrigel substratum showed significant tube formation trends, whereas we observed the tubular networks with the highest density in the groups co-incubated with M@M–Sil-MA_*7Co*_ and M@M–Ag–Sil-MA_*7Co*_. Moreover, these groups exhibited more circles (18.00 ± 4.58% and 18.00 ± 7.00%) and junctions (49.33 ± 9.30% and 49.00 ± 9.00%) relative to those observed in the other groups (Fig. 5h, i).

We then assessed the in vitro ability of the M@M–Ag–Sil-MA hydrogel to promote tissue regeneration via macrophage polarization modulation according to fibroblast migration and tube formation by fibroblasts and endothelial cells. Scratch assays (Fig. 5g) using mouse L929 cells co-incubated with different MCM (days 1 and 7) indicated that L929 cells migrated significantly faster when co-incubated with MCM of M@M–Sil-MA_*7Co*_ and M@M–Ag–Sil-MA_*7Co*_ relative to those incubated with other day-7 and all day-1 MCM, with quantified data (Fig. 5j), clearly demonstrating a migration ratio of 39.88 ± 2.42% for M@M–Sil-MA_*7Co*_ and 41.46 ± 1.50% for M@M–Ag–Sil-MA_*7Co*_ as compared with 6.48 ± 1.08% for Sil-MA_*7Co*_ and 9.57 ± 1.68% for Ag–Sil-MA_*7Co*_ (p < 0.001). To clarify the direct effect of the material on tissue repair, we co-cultured the CM with L929 cells, resulting in detection of no significant cells migration was detected (Additional file 1: Fig. S15). These results indicated that the M@M–Ag–Sil-MA hydrogel may have promoted fibroblast migration through the release of Met and the inherent repair ability of silk fibroin as a result of anti-inflammatory cytokines secreted by M2 macrophage.

These findings revealed that the M@M–Ag–Sil-MA hydrogel system was unable to modulate the anti-inflammatory polarisation of macrophages after a 1-day co-culture, whereas on day 7, we observed altered M2 polarisation. This process is correlated with the dual-controlled release of Met. Moreover, the spatiotemporal modulation of macrophage polarisation mimics the immune response associated with normal wound healing, which avoids the potential risk of infection caused by immune intervention and allows macrophages to promote tissue repair and angiogenesis in a stable, sterile environment.

### Evaluation of inhibited neutrophil extracellular traps (NETs) formation in vitro

The formation of large numbers of NETs in diabetic wounds is a cause of delayed wound healing and leads to persistent inflammation [49]. Both a hyperglycaemic microenvironment and bacterial invasion are potential mechanisms for NETs formation in diabetic wounds [50]. Normal NETs formation is programmed to be pro-inflammatory and bactericidal; however, in the uncontrolled inflammatory microenvironment of diabetic wounds, this process is derailed by the continuous recruitment and infiltration of neutrophils and NETosis [51]. Therefore, inhibition of NETs formation might represent a potential way to promote wound healing, and previous studies report that Met demonstrates significant inhibition of NETs formation [52, 53].

**Fig. 6:**
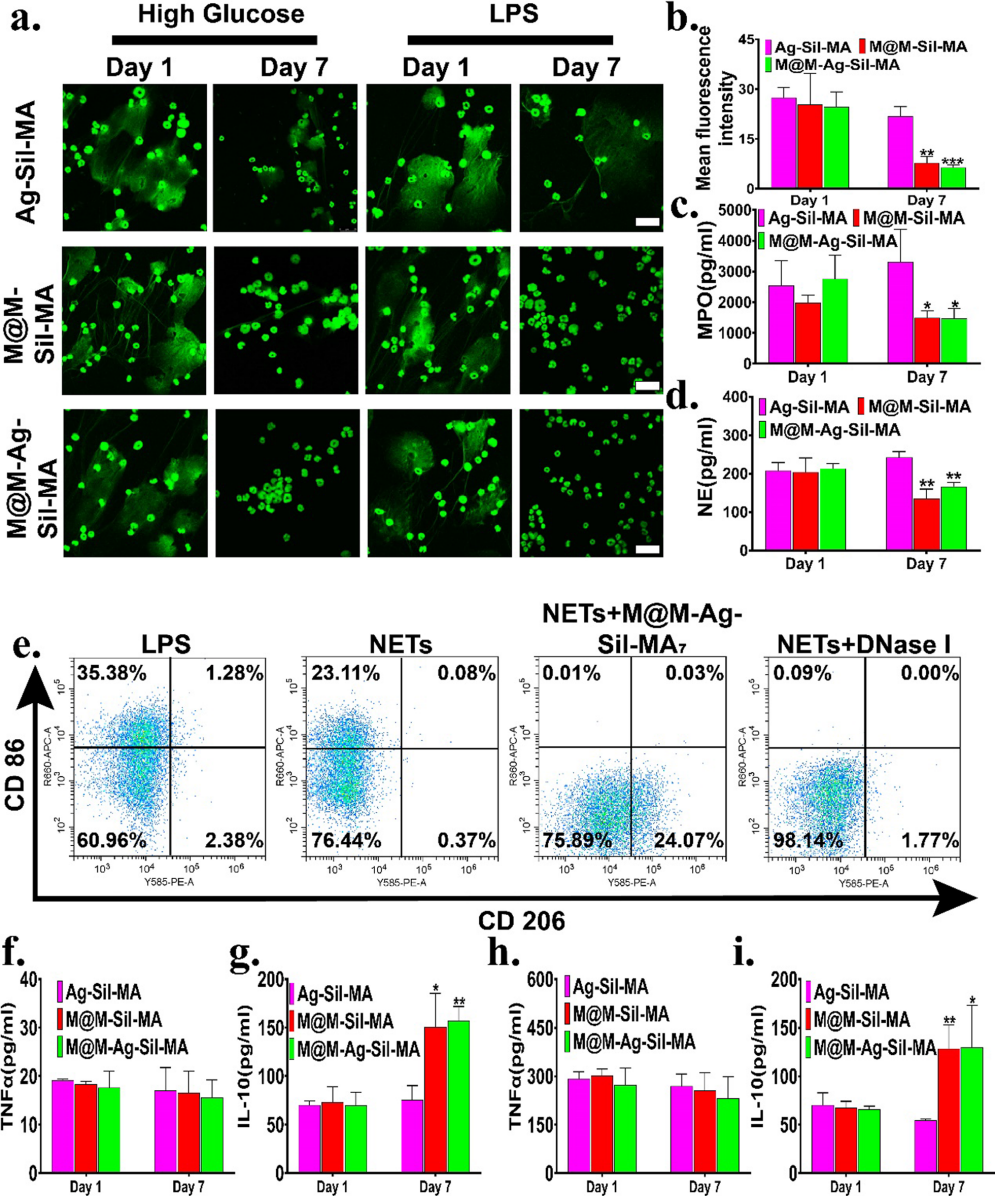
Immunomodulation of NETs in vitro. **a** Fluorescence micrographs of high glucose (50 mM)-treated and LPS-treated neutrophils and stained with SYTOX green on Day 1 and Day 7.The dispersed green area was indicated as NETs (scale bar: 25 μm). **b** Fluorescence intensity of NETs with high glucose-treated neutrophils. **c**, **d** ELISA results for MPO and NE of NETs of high glucose-treated neutrophils. **e** Representative flow cytometry results after co-culture with LPS, extracts of NETs, extracts of M@M-Ag-Sil-MA_7_ (the extracts of M@M-Ag-Sil-MA on day 7), or DNase I of RAW264.7.CD 86 and CD 206 represents M1 macrophages surface markers and M2 macrophages surface markers, respectively. **f**–**i** ELISA results for TNF-α and IL-10 of high glucose-treated and LPS-treated neutrophils

To evaluate the ability of the hydrogel system to inhibit NETs formation, we first established an in vitro model of the NETs formation conditions found in diabetic wounds. Isolated mouse bone marrow neutrophils were activated in vitro by treatment with LPS and high glucose medium (50 mM) to induce NETs formation (Additional file 1: Fig. S16a, b), followed by co-incubation with CM of 1-day cultures of each respective hydrogel system. SYTOX green and immunofluorescence staining to visualise extracellular DNA (Fig. 6a and Additional file 1: Fig. S16c) revealed fluorescence micrographs that showed similar high NETs generation, whereas co-incubation of induced neutrophils (high-glucose/LPS) with CM of M@M–Sil-MA_*7*_ and M@M–Ag–Sil-MA_*7*_ cultures showed a clear reduction in NETs formation. We subsequently confirmed this result by fluorescence intensity quantification (Fig. 6b, Additional file 1: Fig. S17). Furthermore, we observed elevated levels of NETs components, such as neutrophil elastase (NE) and myeloperoxidase (MPO) [54], in all groups incubated with 1-day extracts, whereas these levels were significantly lowered following incubation with M@M–Sil-MA_*7*_ and M@M–Ag–Sil-MA_*7*_ extracts (Fig. 6c, d and Additional file 1: Fig. S18).

To further investigate the effect of hydrogels on macrophage activity via inhibited NETs formation, we co-cultured RAW264.7 cells with medium extracts of NETs formed by high glucose or medium extracts of NETs inhibited by co-incubation with CM of M@M–Ag–Sil-MA_*7*_, with LPS-containing medium and extracts of DNase I-treated NETs serving as controls. Flow cytometry (Fig. 6e) results showed a significant repolarisation to the M1 phenotype after treatment with LPS and high-glucose NETs extract, whereas the M2 phenotype was sustained in the presence of the M@M-Ag-Sil-MA_*7*_-stimulated NETs extract. Surprisingly, we also observed M1 repolarisation in the presence of the DNase I-treated NETs extract, with a possible explanation being that neutrophils also release pro-inflammatory factors during NETosis. Furthermore, ELISA experiments to assess cytokine levels confirmed significant reductions in pro-inflammatory factors, such as TNF-α, and increases in IL-10, a pro-inflammatory factor, following co-incubation with M@M–Ag–Sil-MA_*7*_. These results (Fig. 6f–i) suggested that the M@M–Ag–Sil-MA hydrogel demonstrated a significant immunomodulatory effect on NETs formation via the dual-controlled-release of Met. This regulatory process revealed direct NETs-suppressive and anti-inflammatory activities that sustained the anti-inflammatory polarization of macrophages [55, 56].

### M@M–Ag–Sil-MA hydrogel promotes wound healing in vivo

**Fig. 7:**
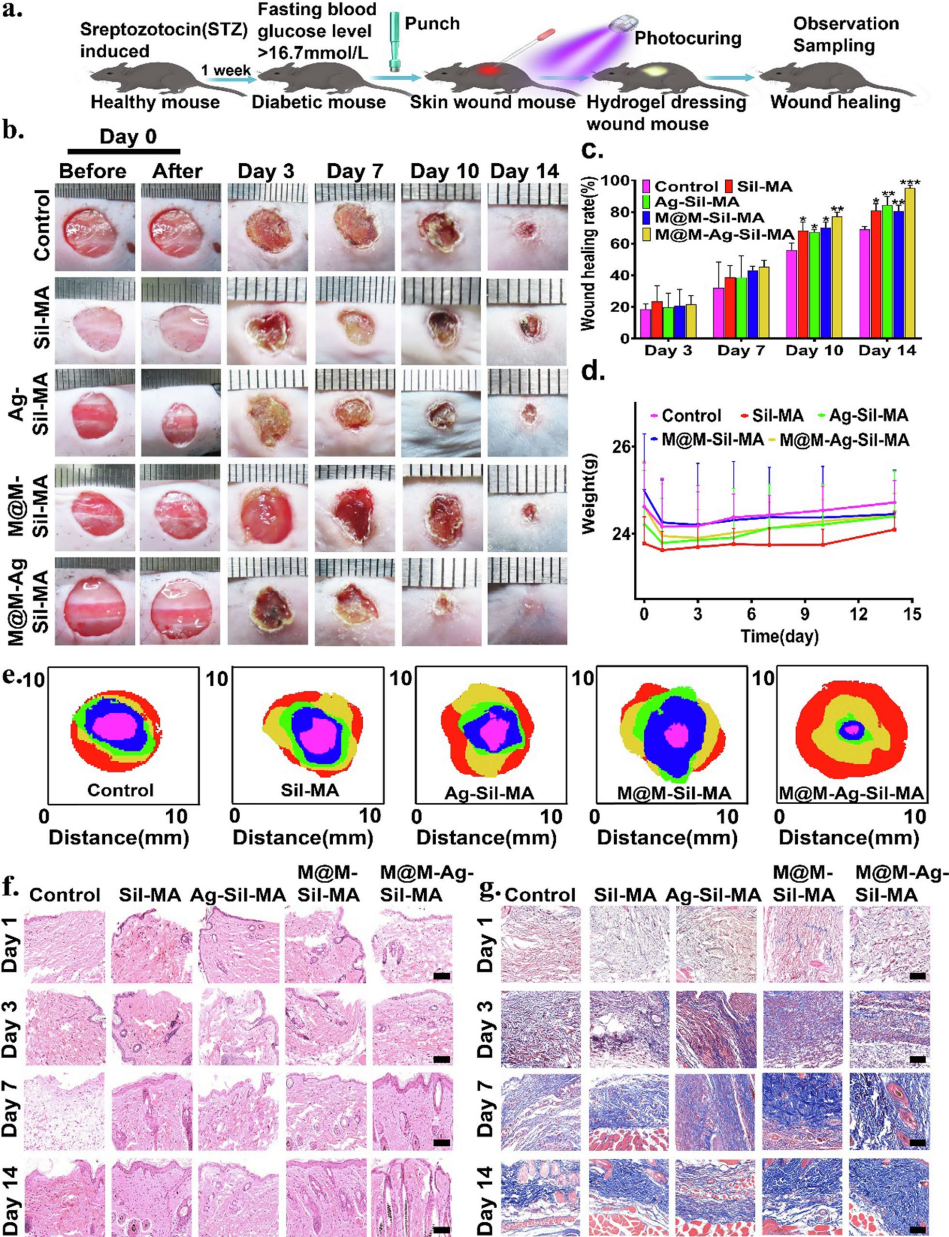
Hydrogel system promoted diabetic wound repair and regeneration in vivo. **a** Schematic diagram of diabetic wound and in situ hydrogel system photocuring. **b** Photographs of the wounds with different treatments on days 0 (before and after photocuring), 3, 7, 10 and 14. **c** Skin wound healing rate of the diabetic wound model at different time points. **d** Weight of the all-diabetic mice at different time points. **e** The overlaid images of the wound healed by different treatments on days 0 (red), 3 (yellow), 7 (green), 10 (blue) and 14 (purple). **f** H&E staining of the wound area on days 1, 3, 7, and 14 reflected the tissue-repair. (scale bar: 50 μm). **g** Masson’s trichrome staining on days 1, 3, 7, and 14 reflected collagen deposition (Scale bar: 50 μm)

Following the establishment of a mouse model of type-2 diabetes (blood glucose level: 16.7 mM) and harbouring a full-sized back wound (diameter: 8 mm) according to previous studies, we injected the respective hydrogel systems (Sil-MA, M@M–Sil-MA, Ag–Sil-MA, and M@M–Ag–Sil-MA) in situ into the wound, followed by UV irradiation at 405 nm to promote photocuring and attachment to the wound surface (Fig. 7a and Additional file 1: Fig. S19). All hydrogel-attached wounds were left uncovered, with saline administration to the wound site used as the control. All wounds were photographed using a digital camera to measure the healing that occurred on days 0, 3, 7, 10, and 14 after surgery, and the gradual shrinkage of wound dimensions was quantified using ImageJ software. The results showed that all wounds to which each of the hydrogels were administered displayed noticeable shrinkage of the wound area during the regeneration stage and relative to the control group (Fig. 7b, c). Specifically, the group administered M@M–Ag–Sil-MA showed the fastest healing rate as compared with all other hydrogel groups. During the early stages of wound healing, M@M–Sil-MA-treated wounds showed marked redness and swelling, which we attributed to direct anti-inflammatory immunomodulation. Similarly, Ag–Sil-MA- and M@M–Ag–Sil-MA-treated wounds exhibited increased drying and shrinkage, possibly due to the inhibition of bacterial infection following the release of Ag NPs and the release of Met, which also reduced scar formation. Meanwhile, we set wound healing using gentamicin-loaded hydrogels as the standard treatment group under the same conditions. At the end point of the experiments, although the wound healing of the positive control group improved relative to the control group, the wound-healing rate remained lower than in the M@M-Ag-SilMA group (Additional file 1: Fig. S20).

Moreover, haematoxylin and eosin (H&E) staining of skin surrounding the wound on days 1, 3, 7, and 14 after surgery showed a markedly higher healing rate in the M@M–Ag–Sil-MA-treated group relative to the other groups (Fig. 7f). Specifically, all groups showed significant inflammatory leukocyte infiltration according to H&E staining on day 1, and the Ag–Sil-MA and M@M–Ag–Sil-MA groups showed significantly better inflammatory cell infiltration on day 3. Notably, H&E staining results on days 7 and 14 clearly showed complete squamous epithelium, well-distributed neovascularisation, new collagen fibre formation, and sebaceous glands in the M@M–Ag–Sil-MA group, indicating the excellent tissue-repair capacity of this hydrogel. To evaluate the collagen formation in different hydrogel-treated wounds, we also performed Masson’s trichrome staining at the same post-surgery time points (Fig. 7g). On days 1 and 3, all wounds harboured granulation tissues with regular re-epithelialisation and few signs of inflammation in M@M–Ag–Sil-MA-treated wounds, whereas on day 14, we observed extensive collagen deposition and dense, wavy collagen fibres in the same wounds along with a more mature newly formed structure that closely resembled normal tissue. Our findings confirmed the inhibition of bacterial invasion, as well as the accelerated granulation tissue formation, dense collagen deposition, and enhanced wound healing efficacy of the M@M–Ag–Sil-MA hydrogel.

### M@M–Ag–Sil-MA hydrogel promotes spatiotemporal immunomodulation in vivo

**Fig. 8:**
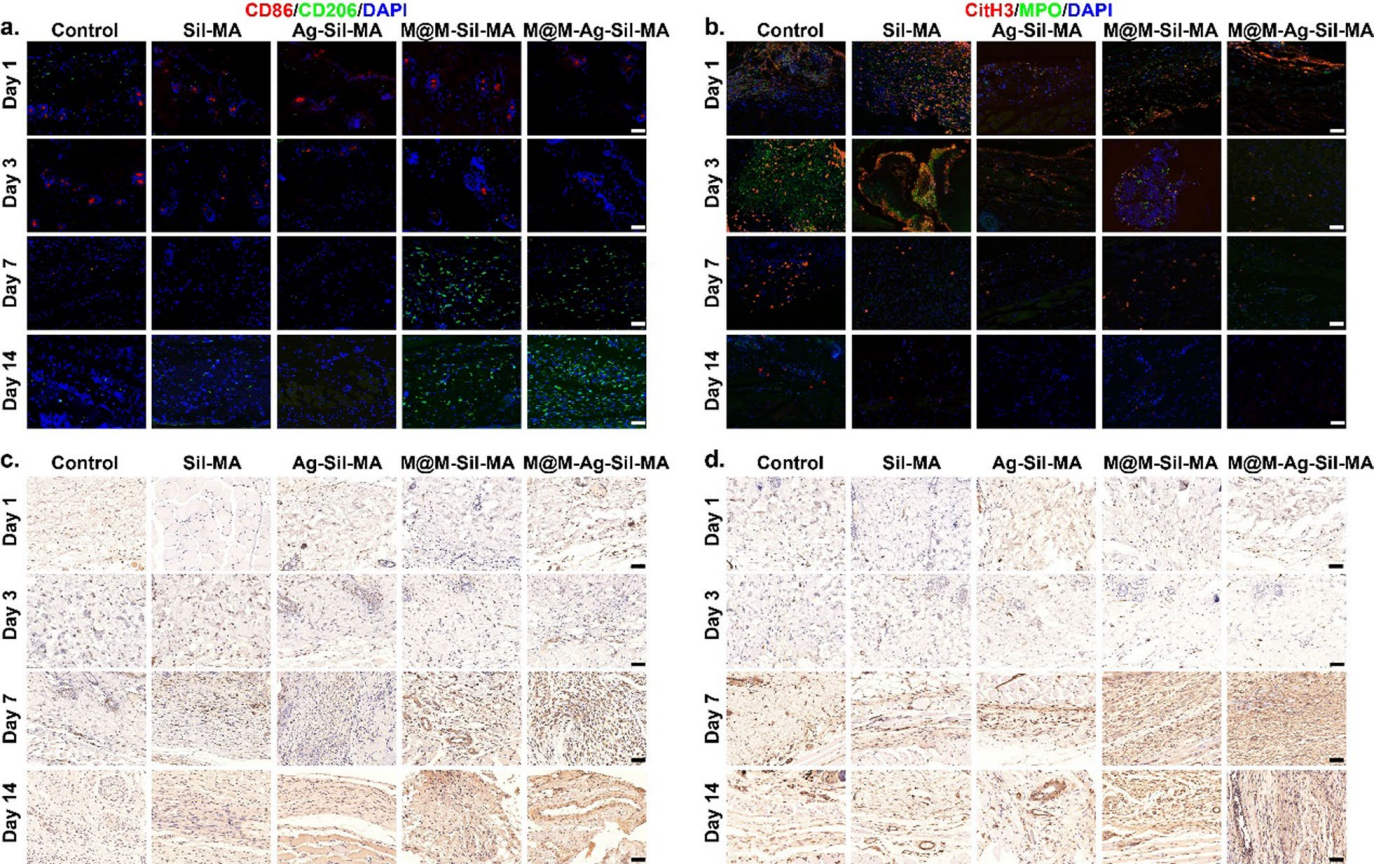
Immunomodulation of macrophages and NETs and tissue regeneration in vivo. **a** Immunofluorescence staining of CD86 (red, M1 macrophage surface marker) and CD206 (green, M2 macrophage surface marker) on days 1, 3, 7, and 14. (Scale bar: 50 μm). **b** Immunofluorescence staining of the markers of NETs formation, CitH3(red) and MPO (green), on days 1, 3, 7, and 14 (Scale bar: 50 μm). **c** CD31 and **d** α-SMA immunohistochemical staining images of the number of CD31-positive and α-SMA -positive cells in the skin tissues of diabetic mice wounds in different treatment groups (Scale bar: 50 μm)

We then performed immunofluorescence staining of wound tissues to evaluate the spatiotemporal immunomodulation of diabetic wounds in vivo using the M@M–Ag–Sil-MA hydrogel. We consistently observed staining of CD86 and CD206 in wounded tissues and representative markers of the pro-inflammatory M1 and anti-inflammatory M2 phenotypes of macrophages (Fig. 8a). On days 1 and 3, we observed a high number of M1 macrophages (CD86+CD206−) in all hydrogel-treated groups, as well as in the control group. Notably, the number of M2 macrophages (CD86−CD206+) in the M@M–Ag–Sil-MA-treated group increased on day 7, whereas tissues from the control and other hydrogel-treated groups showed a persistent pro-inflammatory M1 phenotype, indicating unaltered macrophage polarisation. We continued to observe the same trends on days 14, with the M@M–Ag–Sil-MA-treated group showing a higher density of M2 macrophages than the other groups. However, on day 14, both M1 and M2 macrophage counts in M@M–Ag–Sil-MA-treated wounds decreased relative to those on day 7, which indicated normalised tissue regeneration and decreased involvement of macrophage-mediated tissue repair.

Furthermore, we confirmed the inhibition of NETs formation in M@M–Ag–Sil-MA-treated diabetic wounds by immunofluorescence staining of the NETs components citrullinated histone 3 (CitH3) and myeloperoxidase (MPO) (Fig. 8b). As expected, the M@M–Ag–Sil-MA-treated group showed high NETs formation (CitH3+MPO+) in the first 3 days following surgery and significant NETs inhibition from day 7 onwards, whereas the control and other hydrogel-treated groups maintained high NETs formation and infiltration. Notably, the group treated with M@M–Sil-MA, which also contained Met, did not demonstrate an ability to promote macrophage polarisation to the anti-inflammatory phenotype or inhibit NETs formation in vivo. This might be attributed to a more complicated inflammatory microenvironment of diabetic wounds in vivo, where aggressive anti-inflammatory intervention is incapable of rescuing intrinsic immune functions. These results confirmed that the spatiotemporal immunomodulation by M@M–Ag–Sil-MA was due to its ability to mimic the physiological processes associated with normal wound healing by providing a stable immune microenvironment for angiogenesis and collagen deposition required for tissue regeneration.

### M@M–Ag–Sil-MA hydrogel promotes angiogenesis in vivo

We then determined M@M–Ag–Sil-MA-mediated changes in the rate of angiogenesis in diabetic wounds according to immunohistochemical (IHC) analysis of two markers of vascular formation (VEGF) and α-smooth muscle actin (α-SMA) [57, 58]. IHC staining revealed gradual increases in the number and density of blood vessels in the M@M–Ag–Sil-MA-treated group from days 3 to 14, with both of these measurements higher than those in the control and other hydrogel groups (Fig. 8c, d). Interestingly, we observed high levels of both VEGF and α-SMA during the first 7 days post-surgery in the M@M–Ag–Sil-MA-treated wound, whereas on day 14, there was a slight decrease in VEGF expression. This might be explained by the stabilisation of neovascular alterations during the wound-regeneration process toward the end stages of healing. Furthermore, assessment of in vivo biocompatibility in mice 14 days after surgery by H&E staining of the heart, liver, lungs, spleen, and kidneys confirmed the non-toxicity of the M@M–Ag–Sil-MA hydrogel (Additional file 1: Fig. S21).

## Conclusion

In this study, we synthesized and characterized an injectable M@M–Ag–Sil-MA hydrogel system that can be photocured in situ on diabetic wounds. This M@M–Ag–Sil-MA hydrogel system promotes tissue repair and angiogenesis by first-phase bacterial inhibition followed by the dual-controlled release of Met for spatiotemporal immunomodulation of macrophages and NETs formation. These findings demonstrated that the M@M–Ag–Sil-MA hydrogel system resolved the immune contradiction in diabetic wounds through a two-step spatiotemporal immunomodulation, suggesting its potential as a promising engineered nano-dressing for the treatment of diabetic wounds in orthopaedic surgery (Scheme 1).

## Methods

### Preparation and characterization of MET@MSNs

MET@MSNs were synthesized as previously described [22, 59]. Briefly, MSN (20 mg; XFNANO, Nanjing, China) and Met (60 mg; MACKLIN, Shanghai, China) were dispersed in ethanol and stirred at 37 °C for 24 h. The mixture was centrifuged at 10,000 rpm for 10 min, the supernatant was discarded, and the pellet was resuspended in ethanol and dried at 80 °C for ~ 8 h for further studies. The drug loading (DL%) and encapsulation efficiency (EE%) were determined by UV–Vis spectroscopy (Cary 300; Agilent Technologies, Santa Clara, CA, USA), and the total amount of unloaded MET was calculated according to the absorbance at 234 nm. The results were calculated, as follows:

1 $$\mathrm{DL}(\mathrm{\%})=\frac{Mass \, of \, total \, MET-Mass\, of \,unloaded \,MET\,(Encapsulated \,MET)}{Mass\, of\, total\, MSNs\, and\, encapsulated\, MET}\times 100$$

2 $$\mathrm{EE}(\mathrm{\%}) =\frac{Mass \,of\, total\, MET-Mass \,of\, unloaded \,MET\,(Encapsulated \,MET)}{Mass\, of\, total\, MET}\times 100$$

To determine the particle size and morphology of the MSN and MET@MSNs, SEM was performed (S4800; Hitachi, Tokyo, Japan). The BET method (ASAP2460; Micromeritics, Atlanta, GA, USA) was employed to assess the structural indicators (surface area, pore volume) related to the mesopores of MSN and MET@MSNs. The chemical bond of MSNs and MET@MSNs were analysed using FT-IR (Nicolet IS 10; Thermo Fisher Scientific, Waltham, MA, USA).

### Preparation and characterization of the hydrogel system

The Sil-MA (EFL, Suzhou, China) solution was prepared according to manufacturer instructions. Briefly, Sil-MA (0.5 mg) was dissolved in 0.25% (w/v) initiator LAP solution (5 mL) with stirring at room temperature (23–24 °C) for 30 min. Ag NPs solution (100 ppm; XFNANO) and MET@MSNs with different mass ratios were mixed with Sil-MA solution and vortexed for 5 min, followed by ultasonication (200 W) for 10 min to ensure complete dispersion of the NPs in the Sil-MA solution. The mixture was then irradiated under a 405 nm light source for ~ 25 s to obtain the hydrogel system (Sil-MA, Ag-Sil-MA, M@M-Sil-MA, and M@M-Ag-Sil-MA).

The samples were fixed at the sample stage with conductive glue, and the surface was sprayed with gold. The surface morphology was then observed using SEM. A rotational rheometer was used to test the rheological properties of the hydrogel system (Discovery HR-2; TA instruments, New Castle, DE, USA).

To evaluate the swelling properties of the hydrogel, samples were weighed and the mass was recorded as W_0_. Phosphate-buffered saline (PBS) was added to the samples at different pH values (pH = 6.0, 7.4, and 8.0), after which the samples were removed at a predetermined time point and weighed after absorbing the residual PBS on the surface of the sample with Kimwipes (Kimberly-Clark Corp., Dallas, TX, USA), and the mass was recorded as Wt. The samples were returned to PBS after weighing, and the swelling ratio was calculated, as follows:

$$\mathrm{Swelling \,ratio }\,(\mathrm{\%}) =\frac{Wt-W0}{W0}\times 100$$

Degradation tests (%) were conducted by soaking the samples in PBS at different pH values at 37 °C until complete swelling, and then, the initial mass was recorded as Wi. Then, the samples were placed in PBS solution contained collagenase (Biosharp, Hefei, China) with shaking at 37 °C. At the pre-set time points, the samples were removed and dried with Kimwipes, and the remaining mass was recorded as Wp. The degradation (%) was calculated by the following formula:

$$\mathrm{Degradation }\,(\mathrm{\%}) =\frac{Wi-Wp}{Wi}$$

M@M-Ag-Sil-MA with different mass ratios of Ag NPs and MET@MSNs (1:1, 1:2, and 1:3) was immersed in 2 mL PBS at different pH values (pH = 6.0, 7.4, and 8.0) to evaluate the cumulative release of components. The release system was incubated at 37 °C using a shaker (200 rpm). At specific time points, 1 mL PBS was removed to analyse the concentrations of Ag NPs and Met, after which 1 mL of fresh PBS was added to the release system. Released levels of Ag NPs and Met were determined by measuring the absorbances at 234 nm and 400 nm by UV–Vis spectroscopy, respectively.

### Cells isolated and culture

The femur and tibia medullary cavity of C57/BL6 mice were rinsed with Roswell Park Memorial Institute (RPMI)-1640 medium (Biosharp, Hefei, China) to obtain primary cells. Neutrophils were isolated using the EasySep mouse neutrophil enrichment kit (Stemcell Technologies, Vancouver, BC, Canada) according to manufacturer instructions and cultured in RPMI-1640 medium supplemented with 10% foetal bovine serum (FBS; Gibco, Gaithersburg, MD, USA) and 0.1 mg/mL primocin (InvivoGen, San Diego, CA, USA).

EA.hy926, L929, and RAW264.7 cells were incubated in high-glucose Dulbecco’s modified Eagle medium (DMEM; Gibco) supplemented with 10% FBS, 100 U/mL penicillin, and 100 μg/mL streptomycin.

### Verification of neutrophil cells

The obtained neutrophils were collected through centrifugation at 300*g* for 10 min at 4 ℃, and the pellets were resuspended with 100 μL PBS containing 0.25 μg of the allophycocyanin (APC)-conjugated anti-mouse/human CD11b antibody (Biolegend, San Diego, CA, USA) and the phycoerythrin (PE)-conjugated anti-mouse Ly-6G antibody (Biolegend), followed by incubation on ice for 30 min. Levels of CD11b and Ly-6G in the neutrophils were analysed by flow cytometry using the CytoFLEX system (Beckman Coulter, Pasadena, CA, USA).

### Evaluation of in vitro cytotoxicity

RAW264.7, EA.hy926, and L929 cells were used to evaluate in vitro cytotoxicity. M@M-Ag-Sil-MA and the three cells were co-cultured in 6-well plates at 37 ℃, after which M@M-Ag-Sil-MA and the medium were removed and replaced with DMEM containing 10% Cell Counting Kit-8 reagent (Biosharp). After incubating for 2 h, the absorbance was measured at 450 nm using a microplate reader (Epoch; BioTEK, Winooski, VT, USA).

Additionally, RAW264.7 cells were stained using the LIVE/DEAD cell imaging kit (Invitrogen, Carlsbad, CA, USA) for 15 min, and EA.hy926 cells were subsequently stained with 100 nM TRITC-phalloidin (Yeasen, Shanghai, China) and 4′,6-diamidino-2-phenylindole (DAPI, Biosharp) to observe cell morphology. Stained cells were observed under an inverted fluorescence microscope (ECLIPSE Ts2; Nikon, Tokyo, Japan).

### CM and MCM preparation

Briefly, four samples including Sil-MA, M@M-Sil-MA, Ag-Sil-MA, and M@M-Ag-Sil-MA were soaked with PBS with shaking at 37 °C. Based on the observed release of Met and Ag NPs, the PBS solution was collected on days 1 and 7, centrifuged, filtered using a 0.22-μm filter, and mixed with DMEM at a ratio of 1:2 (v/v). This CM was then prepared for further testing. For MCM preparation, macrophages were cultured with CM for 24 h, followed by replacement of CM with complete medium (DMEM). After another 24-h incubation, the medium was collected to prepare MCM using the same methods as those for CM.

### NETs formation and extraction

Round coverslips were placed on 12-well plates, onto which isolated neutrophils were seeded (1 × 10^5^cells/well) in RPMI-1640 medium containing phorbol (Sigma-Aldrich, St. Louis, MO, USA) and incubated for 2 h at 37 °C. The cells were then fixed with 4% paraformaldehyde for 15 min and washed three times with PBS. The NETs were stained with 0.5 μM SYTOX Green nucleic acid stain (Invitrogen) and observed using confocal laser scanning microscopy (LSM710; Carl Zeiss, Oberkochen, Germany). NETs extracts were collected using the same methods as those for CM collection.

### In vitro antibacterial test

*S. aureus* (ATCC43300) and *E. coil* (ATCC 35218) cultured in tryptic soy broth were used for antibacterial tests. Bacterial suspensions (1 × 10^7^ CFU/mL) were spread onto Mueller–Hinton agar plates. Sil-MA containing different materials (Sil-MA, M@M-Sil-MA, Ag-Sil-MA, or M@M-Ag-Sil-MA) were photocured on curing rings, which were then placed on the agar plates containing the bacteria and incubated 24 h at 37 °C. The diameter of the inhibition zone around the samples was subsequently measured.

The different CMs (Sil-MA, M@M-Sil-MA, Ag-Sil-MA, and M@M-Ag-Sil-MA) were co-cultured with bacterial suspension (1 × 10^7^ CFU/mL) and incubated at 37 ℃, with the control group treated with PBS. At a predetermined time point, 1 mL of the suspension was used to generate a tenfold gradient dilution, with 100 μL of the dilution spread onto sheep blood agar plates. After overnight incubation at 37 °C, bacterial colonies on the plates were counted.

To observe bacterial morphology, sterile titanium sheets, different CM, and bacterial suspension were co-cultured in 6-well plates 24 h at 37 °C, after which the titanium sheets were subjected to SEM analysis. Briefly, the sheets were fixed with 2.5% glutaraldehyde overnight at 4 °C, and samples were dehydrated using an ethanol gradient (50%, 70%, 80%, 90%, 95%, and 100%) for 10 min at room temperature (23–24 °C). After freeze-drying, the surface of the sheets was sprayed with gold, followed by SEM analysis.

### Flow cytometry

RAW264.7 cells were seeded onto 6-well plates at a concentration of 2 × 10^5^cells/well. After incubation for 24 h at 37 ℃, the medium was removed and washed three times with PBS, followed by the addition of different CM to each well. To mimic the in vivo inflammatory conditions of diabetic wounds and explore the effect of NETs and DNase I on macrophage repolarization, LPS (Sigma-Aldrich), NETs extracts, and DNase I (Sigma-Aldrich) were added into each well, respectively. After a 24-h culture, RAW264.7 cells were collected via centrifugation at 1000 rpm for 5 min, and the pellets were resuspended with 100 μL PBS containing 0.25 μg APC-conjugated anti-mouse CD86 antibody (Biolegend) and 0.5 μg PE-conjugated anti-mouse CD206 antibody (Biolegend), followed by incubation on ice for 30 min. CD86 and CD206 levels on RAW264.7 cells were analysed by flow cytometry using the CytoFLEX system (Beckman Coulter).

### RT-PCR

RAW264.7 cells treated with different CM for 24 h were evaluated for iNOS and Arg-1 expression by RT-PCR. Total RNA was extracted and purified using the EZ-press RNA purification kit (EZBioscience, Roseville, MN, USA) and then reverse transcribed into cDNA using a Colour reverse transcription kit (EZBioscience). Quantitative RT-PCR was performed using 2 × Colour SYBR Green qPCR master mix (EZBioscience) and relative expression was calculated using the 2^−ΔΔCt^ method. Primer sequences for glyceraldehyde 3-phiosphate dehydrogenase (Gapdh), iNOS, and Arg-1 are shown in Additional file 1: Table S1.

### ELISA

RAW264.7 cells treated with different CM for 24 h, and the medium was collected and centrifuged at 3000 rpm for 20 min. The supernatant was then used to determine inflammatory cytokine expression using ELISA Kits (Dakewe Biotech, Guangzhou, China) according to manufacturer instructions.

### Tube formation assay

Briefly, EA.hy926 cells (1 × 10^4^ cells/well) pre-treated with different MCM for 24 h were seeded onto μ-slide plates (IBIDI GmbH, Munich, Germany) pre-coated with Matrigel matrix (BD Biosciences, Franklin Lakes, NJ, USA). After incubation for 6 h at 37 °C, the formed tubes were fixed and stained with 100 nM fluorescein isothiocyanate-phalloidin (Yeasen) and observed using an inverted fluorescence microscope. The numbers of junctions and circles were counted manually.

### Scratch assay

L929 cells (2 × 10^5^cells/dish) were seeded in 35-mm cell culture dishes and incubated at 37 ℃ to 90% confluence. Subsequently, 200-μL pipette tips were used to draw a line at the bottom of the dishes, which were then washed three times with PBS. The cells were then co-cultured with MCM in five groups for 24 h at 37°. At 0 h and 24 h, the cells in each group were fixed, washed, stained with Crystal Violet for 3 min, and observed using an optical microscope. Cell-migration rates in each group were assessed using ImageJ software (v1.52; NIH, Bethesda, MD, USA).

### Inhibition of NET formation

To explore Met inhibit NETs formation in the infective microenvironment of diabetic wounds, neutrophils were cultured with CM in three groups containing glucose or LPS for 2 h, after which the cells were stained with 1 μM SYTOX Green (Invitrogen) and observed by confocal laser scanning microscopy.

### Streptozotocin (STZ)-induced diabetic mice

All animal experiments were approved by the Animal Welfare Ethics Committee of The First Hospital Affiliated University of Science and Technology of China. C57BL/6 mice (6–8 weeks, 23–26 g) were used to induce diabetes. Briefly, 100 mg/kg STZ (Sigma-Aldrich) was injected intraperitoneally into mice fasted from food and water for 1 day prior to injection. Blood glucose levels were determined using glucose meters (Roche, Penzberg, Germany), and all mice with glycaemia (≥ 16.7 mM) were considered diabetic.

### Evaluation of in vivo wound healing

We randomly divided 45 diabetic mice into five groups: control, Sil-MA, M@M-Sil-MA, Ag-Sil-MA, and M@M-Ag-Sil-MA. For consistency in animal experiments, each group contained 9 mice. These diabetic mice were anesthetized by inhalation of CO_2_. After shaving and disinfecting the dorsal skin of the mice, a pouch with a diameter of 8 mm was used to create a skin wound, after which 0.2 mL of hydrogel (Sil-MA, M@M-Sil-MA, Ag-Sil-MA, or M@M-Ag-Sil-MA) was dropped onto the wound and photocured under 405 nm UV light for 25 s. On days 0, 3, 7, 10, and 14 after surgery, the weight of the mice was obtained, and the condition of the wounds were recorded to assess the wound-healing rate.

### Histological analysis

On days 1, 3, 7, and 14 after surgery, random mice from each group were euthanized. The wounded skin tissues were collected and fixed in 4% paraformaldehyde, dehydrated using an ethanol gradient, embedded in paraffin wax, and cut into sections using an RM2016 microtome (Leica, Wetzlar, Germany). Sections were stained with H&E (Solarbio, Beijing, China) and Masson’s trichrome (Solarbio) to assess the degree of inflammatory cell infiltration, collagen deposition, and bacterial infection. For immunofluorescence staining, primary CD86 (1:3000; Bioss, Beijing, China), CD206 (1:400; Servicebio, Wuhan, China), MPO (1:400, Servicebio), CitH3 (1:3000; Abcam, Cambridge, UK), secondary horseradish peroxidase (HRP)-conjugated goat anti-rabbit IgG (H+L) (1:500; Servicebio), and Alexa Fluor 488-conjugated goat anti-rabbit IgG (H+L) (1:400; Servicebio) antibodies were used to observe the degree of inflammatory response, macrophage phenotype, and NETs in wounded skin tissues. To observe angiogenesis in wound skin tissues, sections were incubated with primary antibodies for IHC analysis [mouse anti-α-SMA (1:300; Bioss) and rabbit anti-VEGF antibody (1:200, Bioss, China)] at 4 ℃ overnight, followed by incubation with the secondary antibodies HRP-conjugated goat anti-mouse IgG (H+L) (1:200; Servicebio) and HRP-conjugated goat anti-rabbit IgG (H+L) (1:200; Servicebio) for 1 h. The binding sites were visualized with a 3,3′-diaminobenzidine detection kit (DAKO, Glostrup, Denmark), counterstained with haematoxylin (Servicebio), and mounted with neutral resinto (Servicebio). All stained sections were observed using a microscope (Ci-S; Nikon).

### Evaluation of in vivo biocompatibility

The major organs (heart, liver, spleen, lung, and kidney) from each group were collected, fixed, dehydrated, embedded, cut, and stained with H&E. The sections were then observed using an optical microscope to assess the in vivo biocompatibility of the samples.

### Statistical analysis

All data were exhibited as the mean ± standard deviation and analysed using GraphPad Prism software (version 8.0) and Origin (Version 2019b). Statistically significant values were assessed using two-sided student’s t test and one-way analysis of variance (ANOVA) test. P value < 0.05 was considered statistically significant.

## Supplementary Information

**Additional file 1.** Additional figures and Tables.

## Data Availability

The datasets used and/or analysed during the current study are available from the corresponding author on reasonable request.

## Abbreviations

α-SMA: α-Smooth muscle actin
Ag: Silver
BET: Brunauer–Emmett–Teller
CitH3: Citrullinated histone 3
CM: Conditioned medium
MCM: Macrophage-conditioned medium
ELISA: Enzyme-linked immunosorbent assay
IHC: Immunohistochemical
IL: Interleukin
LPS: Lipopolysaccharide
Met: Metformin
MPO: Myeloperoxidase
MSN: Mesoporous silica microsphere
NET: Neutrophil extracellular trap
NP: Nanoparticle
ROS: Reactive oxygen species
SEM: Scanning electron microscopy
Sil-MA: Methacryloxylated silk fibroin hydrogel
TNF: Tumour necrosis factor
ZOI: Zone of inhibition
