# Supplementary material for: An injectable photo-cross-linking silk hydrogel system augments diabetic wound healing in orthopaedic surgery through spatiotemporal immunomodulation
Source: J Nanobiotechnology. 2022 May 14;20:232. doi: 10.1186/s12951-022-01414-9 (PMC9107711; doi:10.1186/s12951-022-01414-9)
Supplement: Supplementary file 1 — Additional file 1. Additional figures and Tables. [file 12951_2022_1414_MOESM1_ESM.pdf]

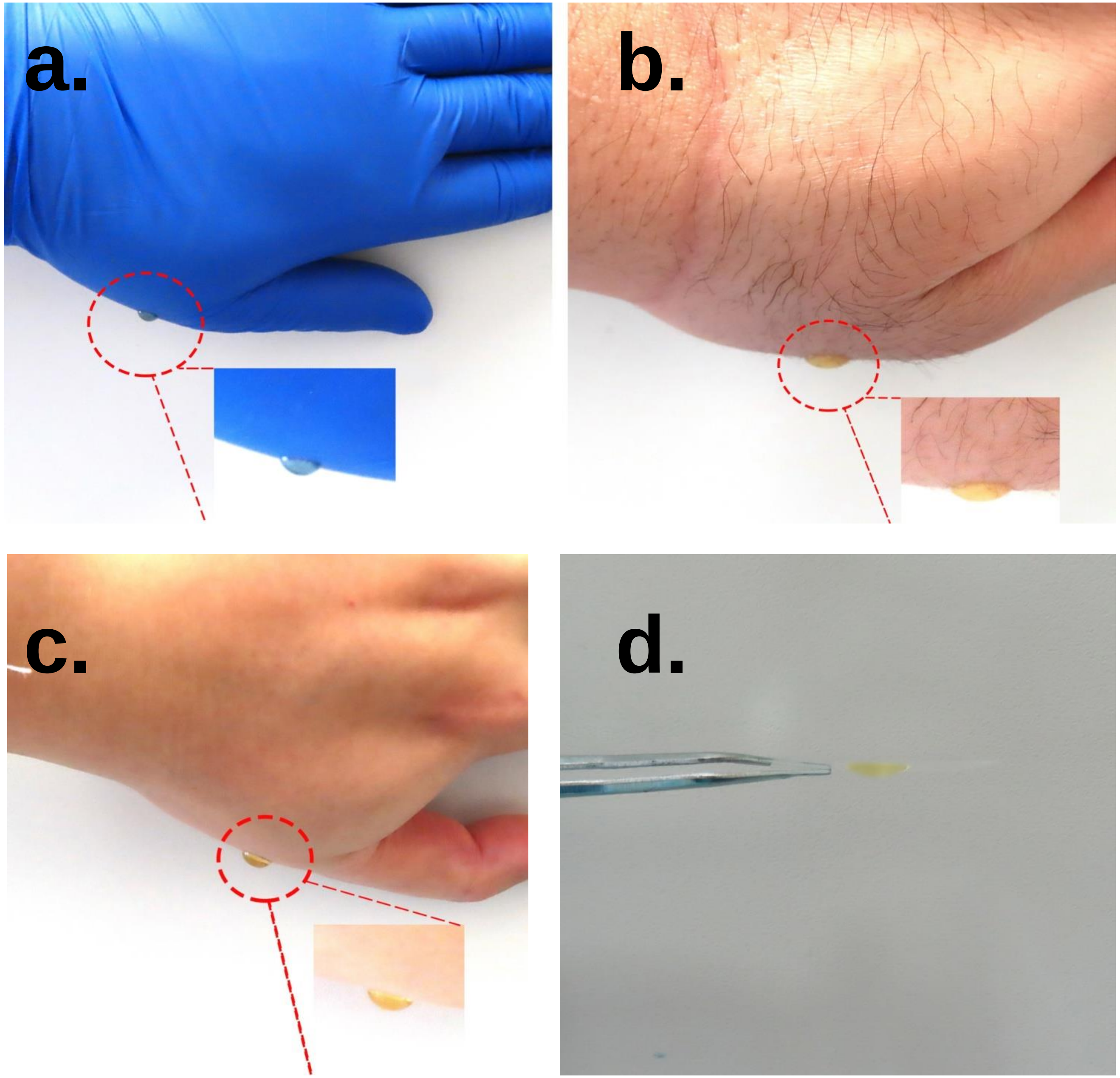

Fig S1. Adhesion of Sil-MA to different materials a). Nitrile, b,c). Skin with or without hair, d). Glass at the inverted direction.

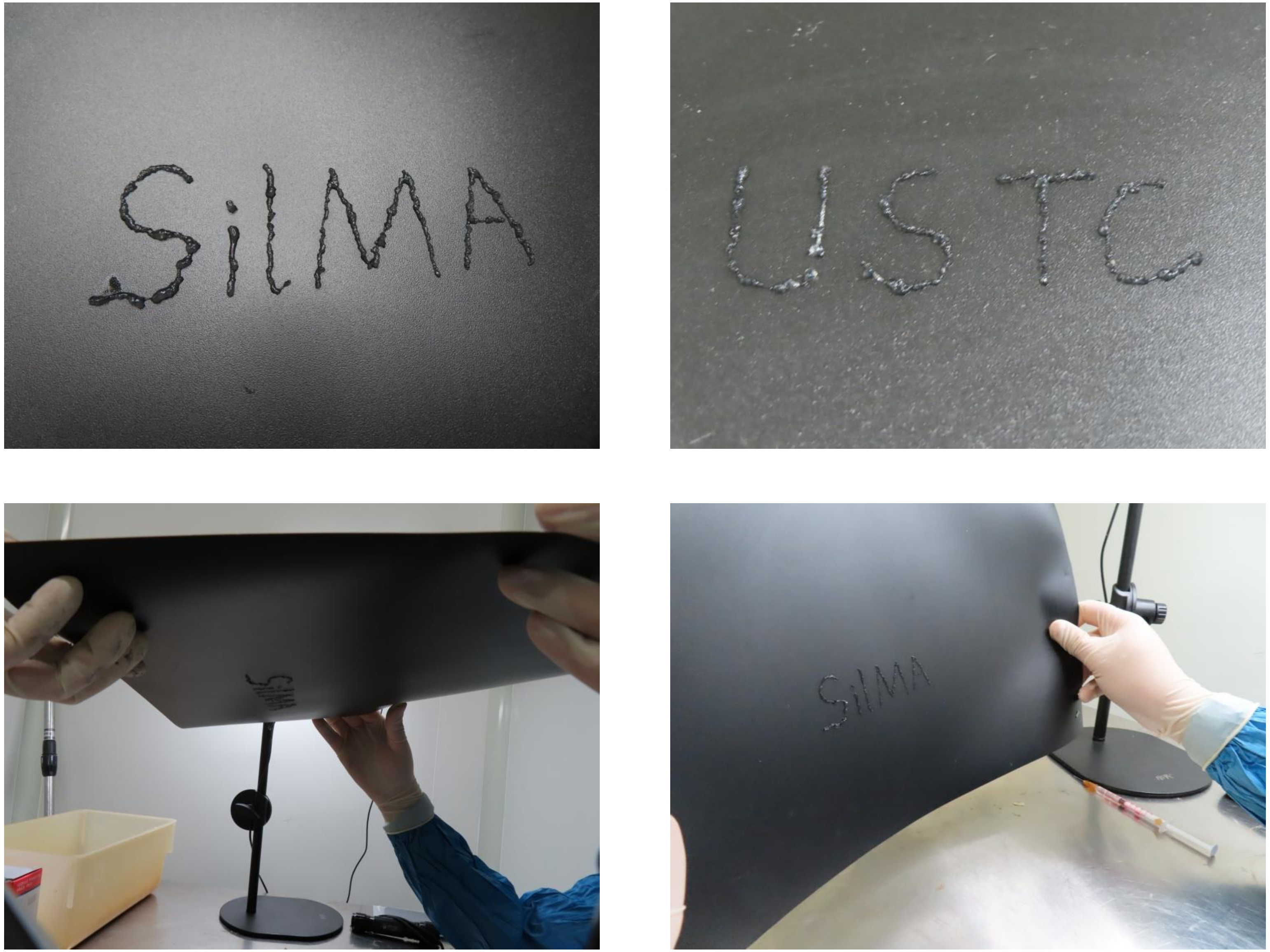

Fig S2. The writing ability and adhesion properties of the Sil-MA.

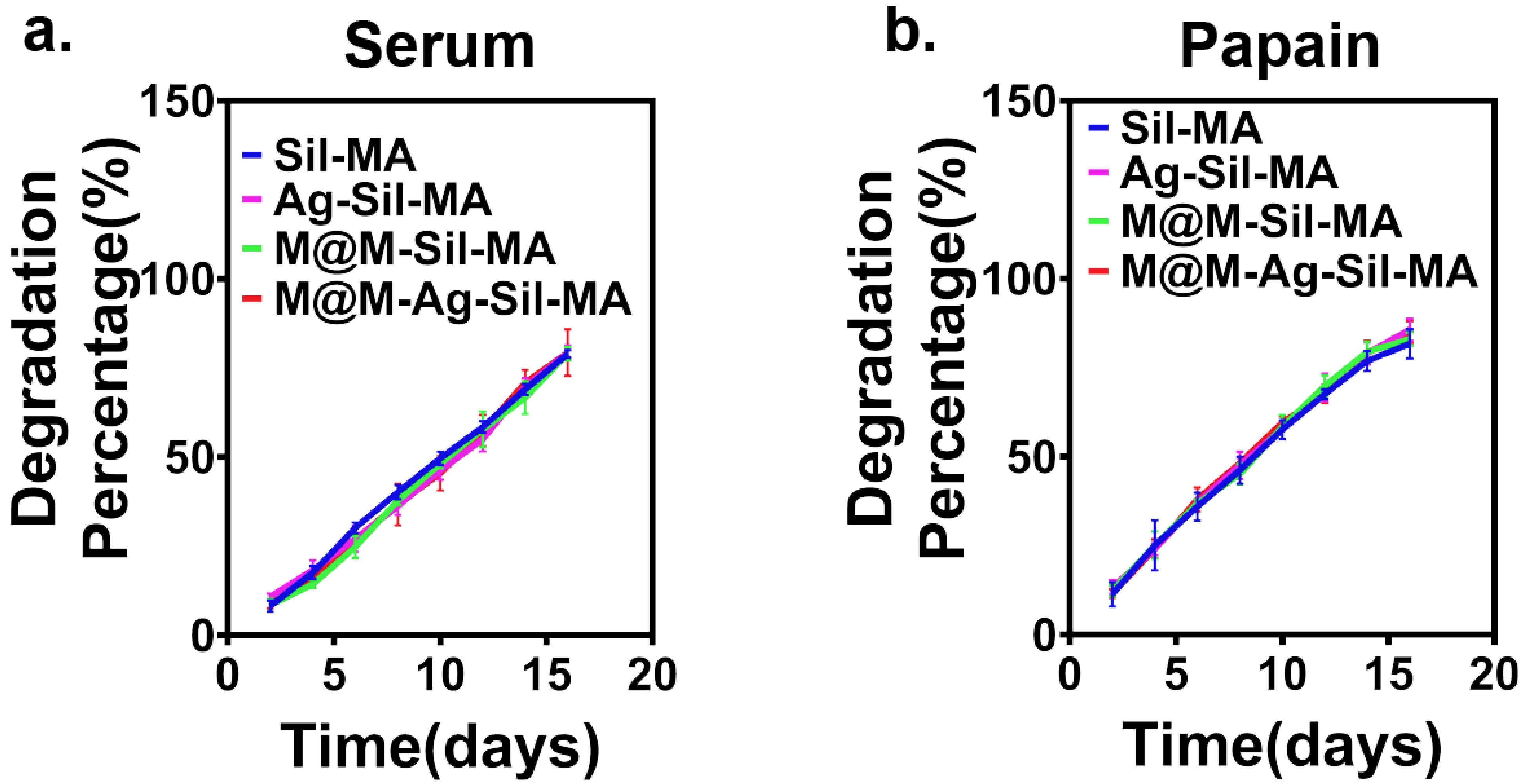

Fig S3 Degradation property of the different hydrogel system soaked into a). serum and b). papain until complete swelling.

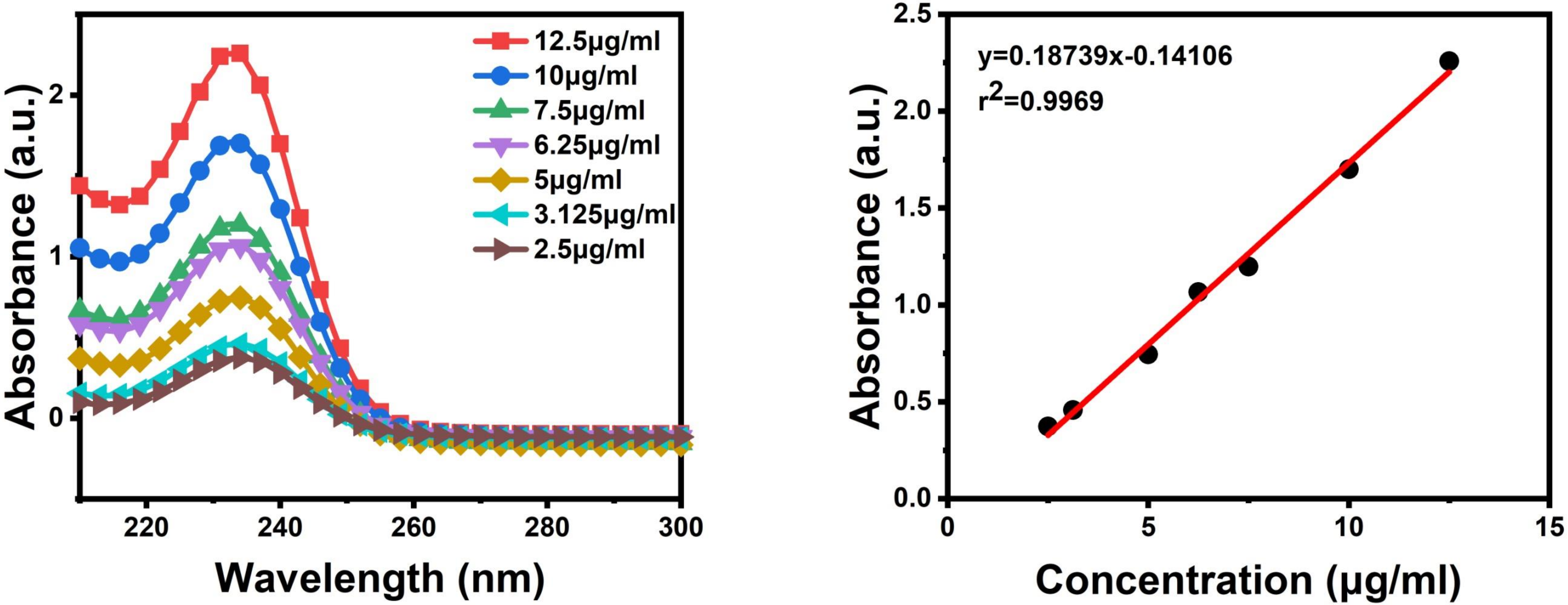

Fig S4. The standard curve of metformin at 234nm.

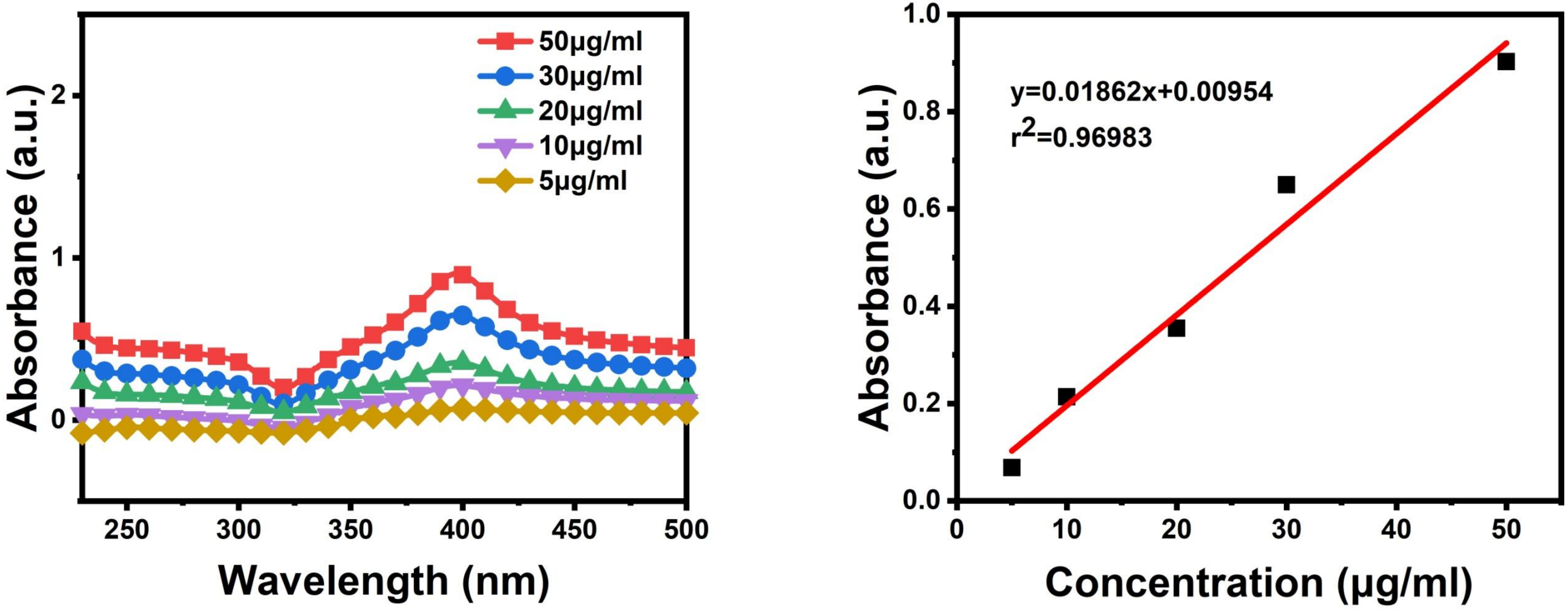

Fig S5. The standard curve of Ag NPs at 400nm.

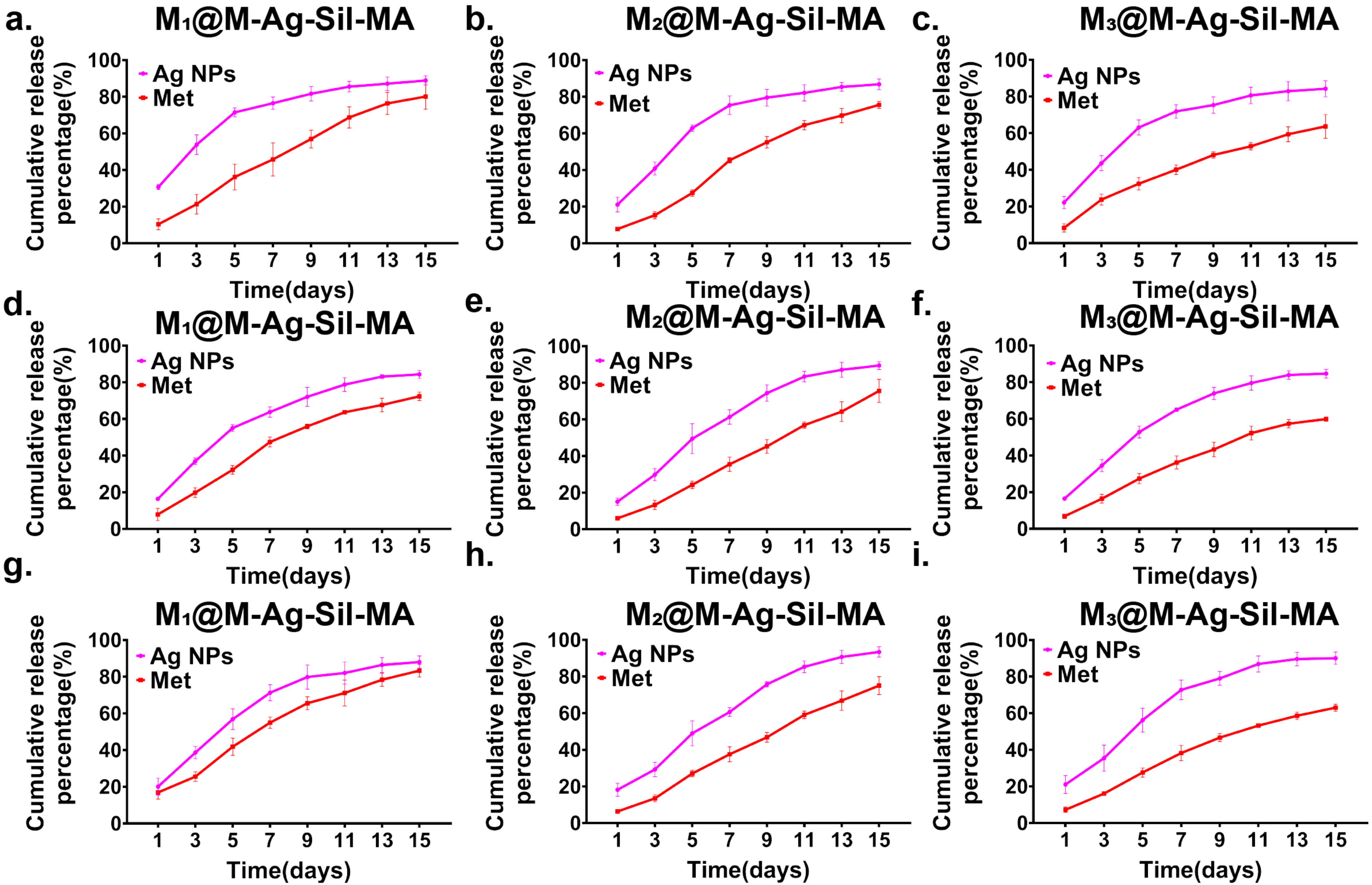

Fig S6. Drug release assay of the hydrogel system in vitro. Cumulative release percentage curves of Met and Ag NPs of hydrogel systems with different MET@MSNs andAg NPs mass ratios (1：1, 2:1, and 3:1) in 2 ml neural (pH= 7.4) (a-c), acidic (pH=6.0) (d-f)and alkaline(pH=8.0) (g-i) PBS at 37℃using a shaker (200 rpm).

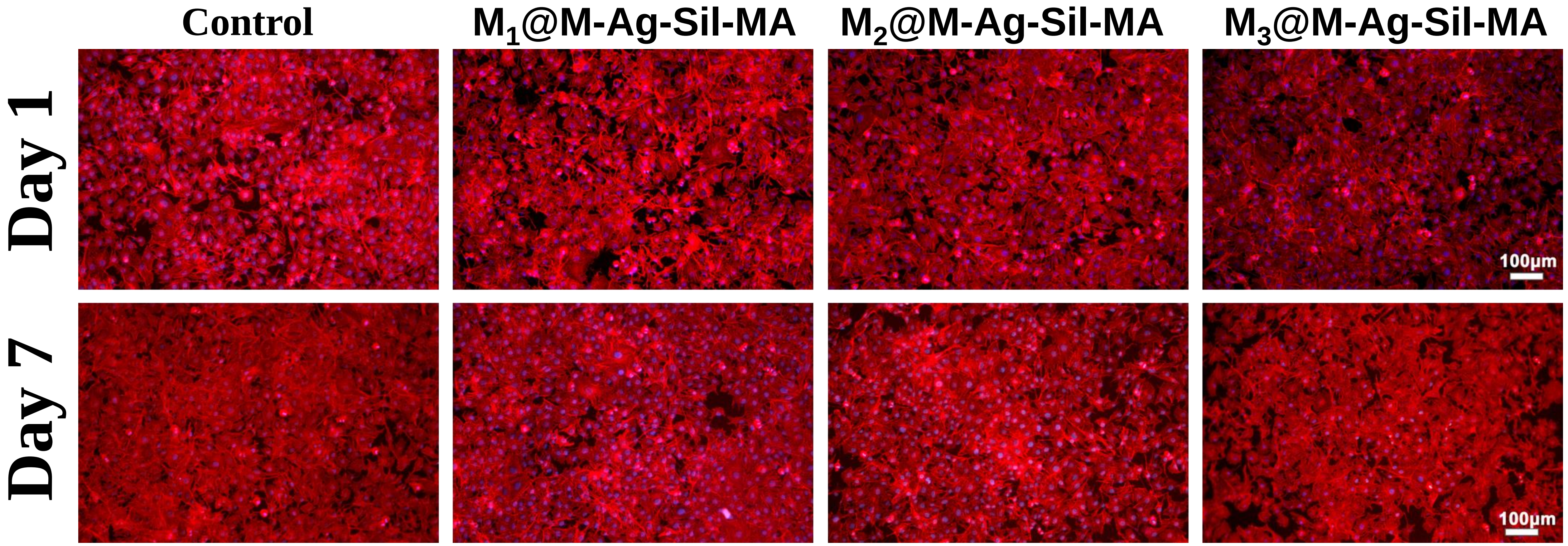

Fig S7. Representative fluorescence microscopy of Ea.hy 926 cells on Day 1 and Day 7.

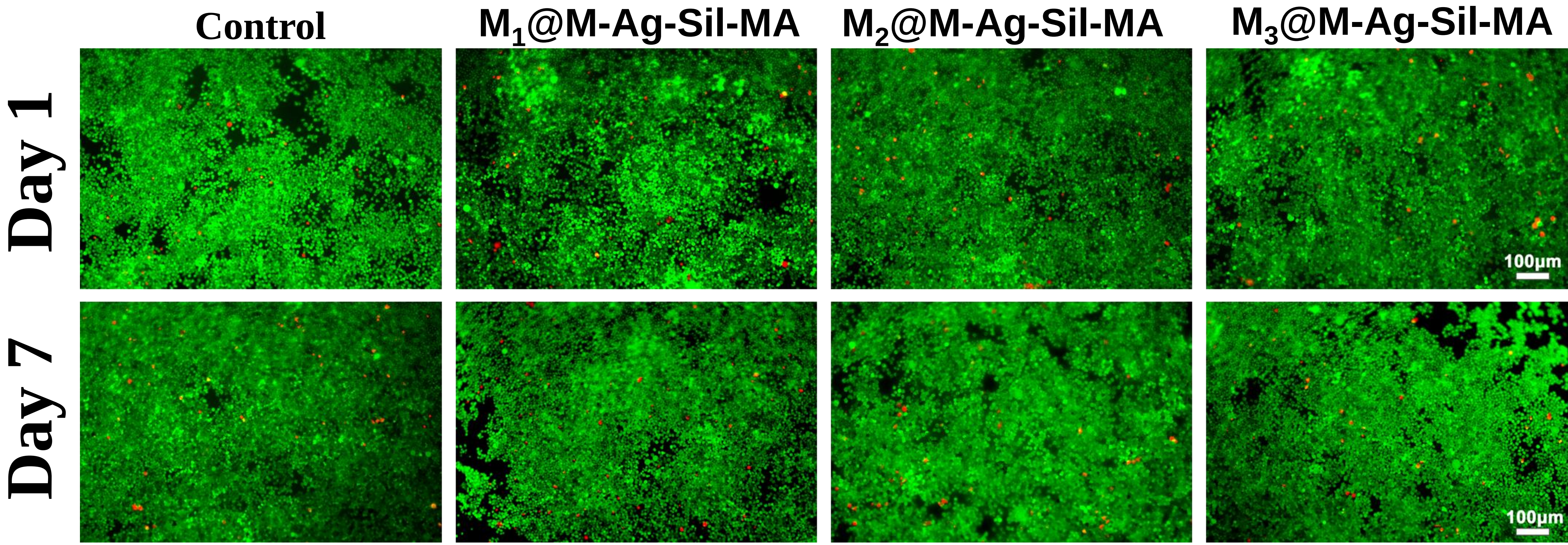

Fig S8. Live/Dead assay of RAW264.7 on days 1 and 7 (Scale bar: 100μm).

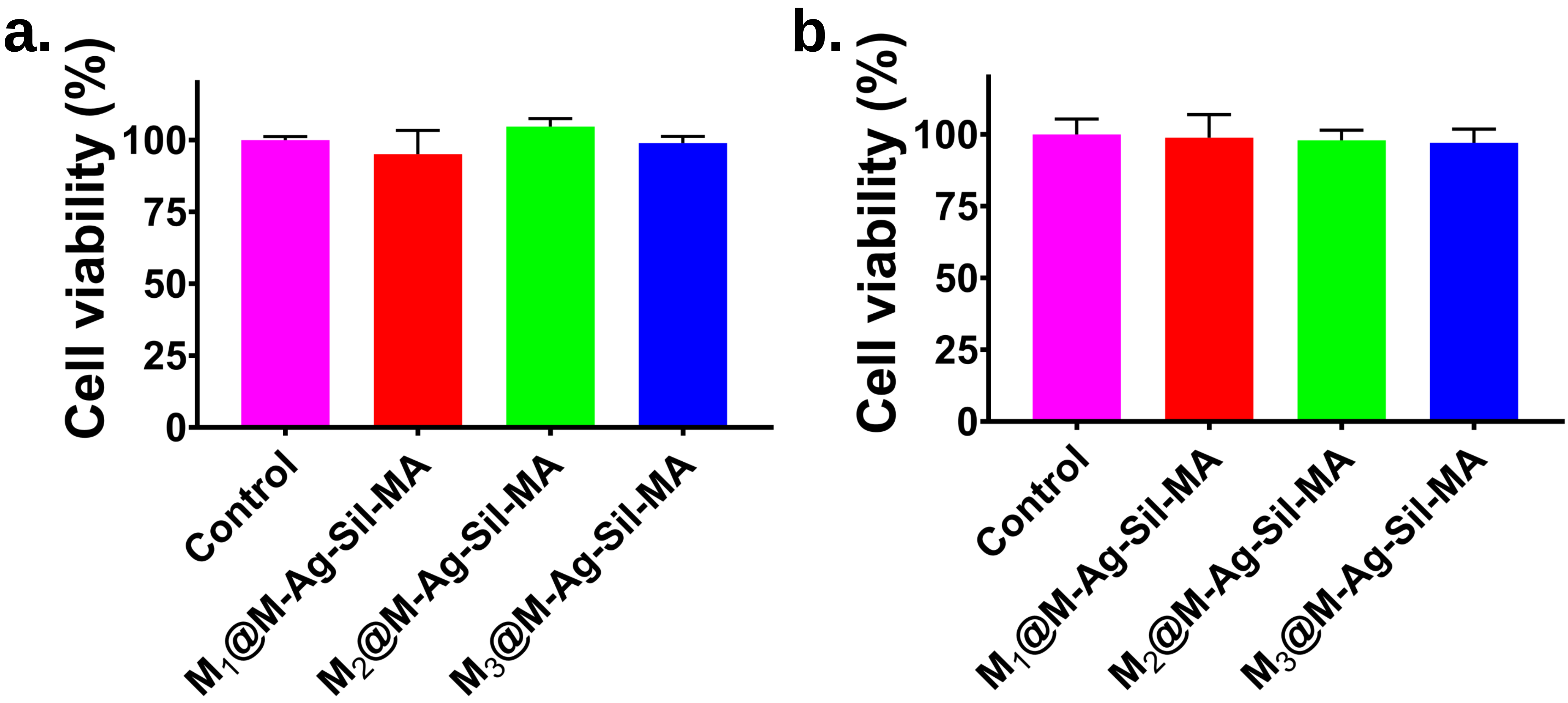

Fig S9. Biocompatibility of L929 cells by CCK8 assay on days a). 1 and b). 7

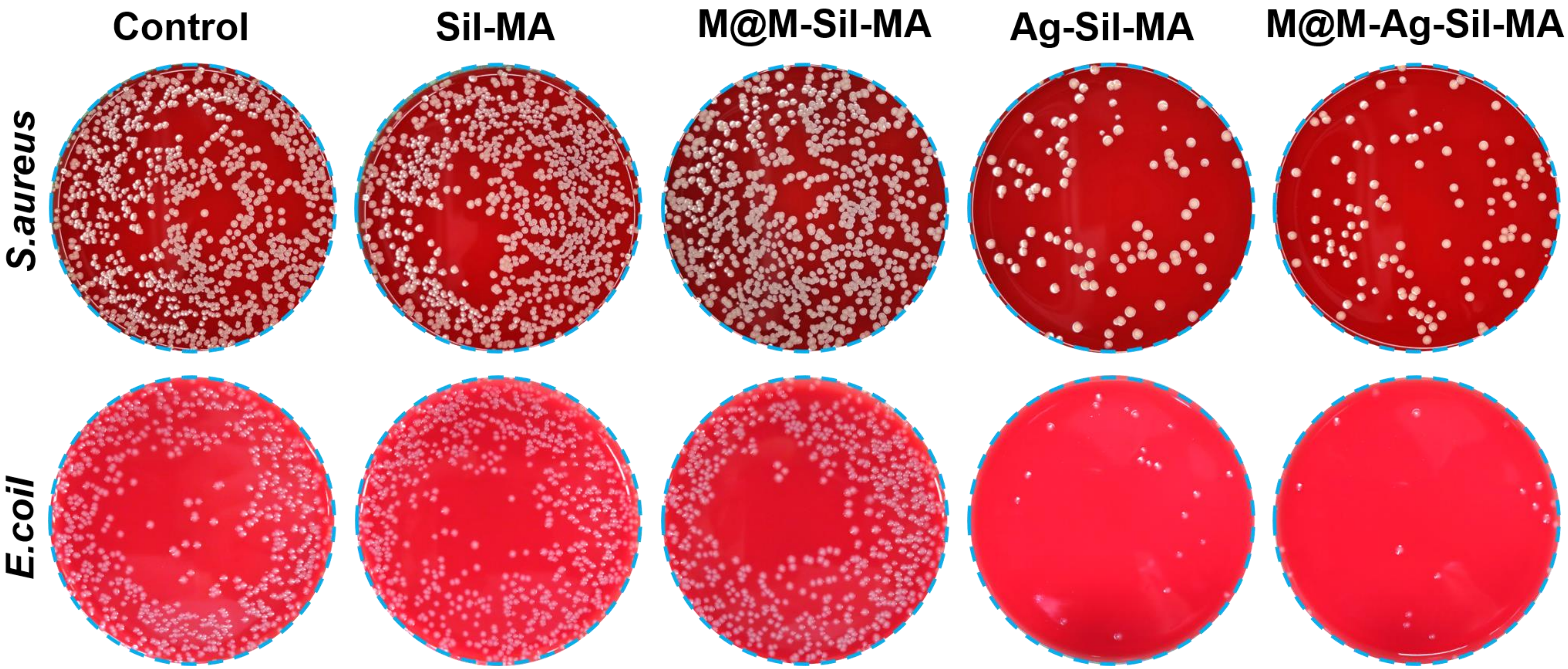

Fig S10. Representative culture images of bacterial colonies at day 7.

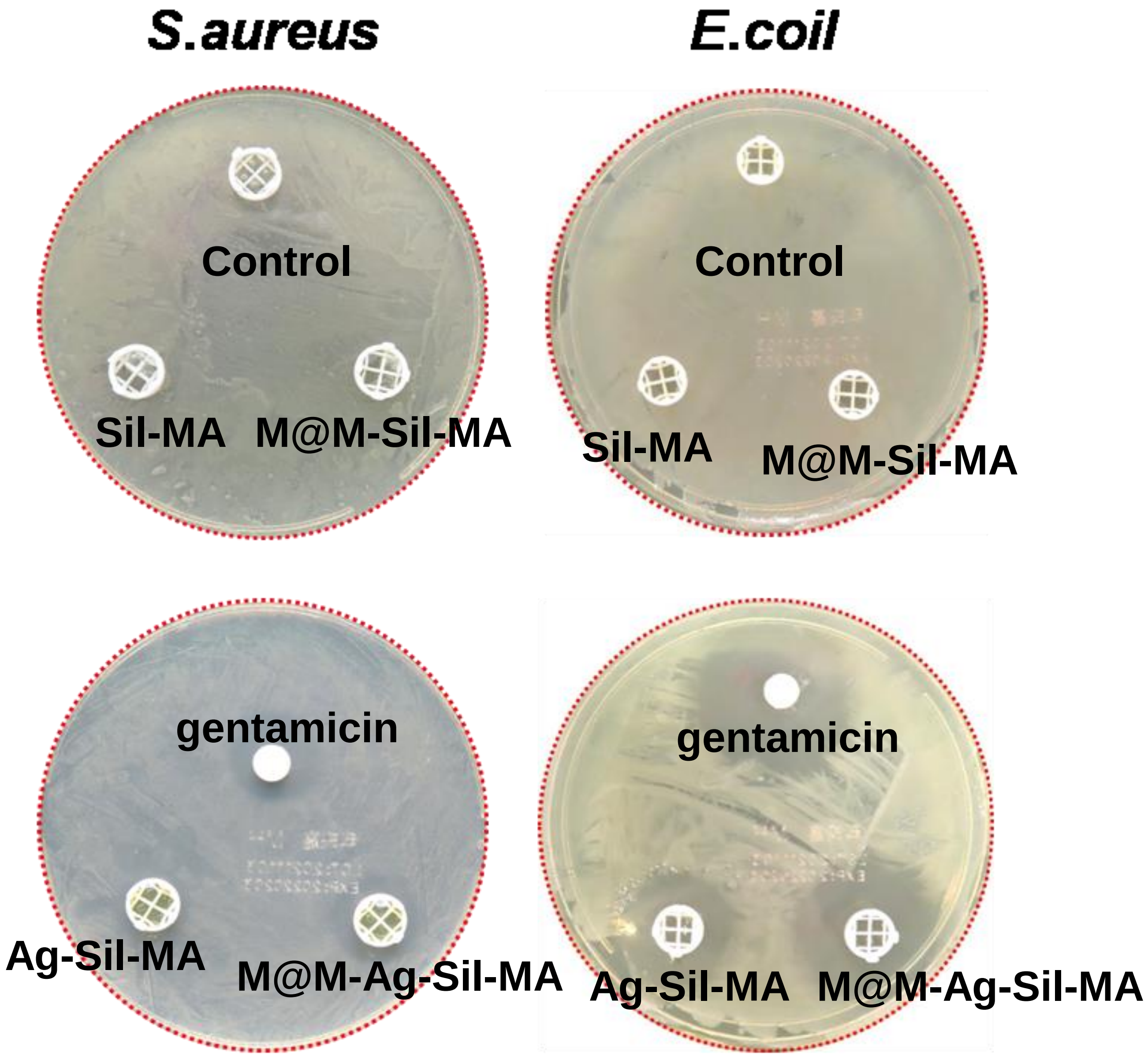

Fig S11. Representative photos of the hydrogel systems and gentamicin disks of ZOI against *S. aureus* and *E. coli* at day 1.

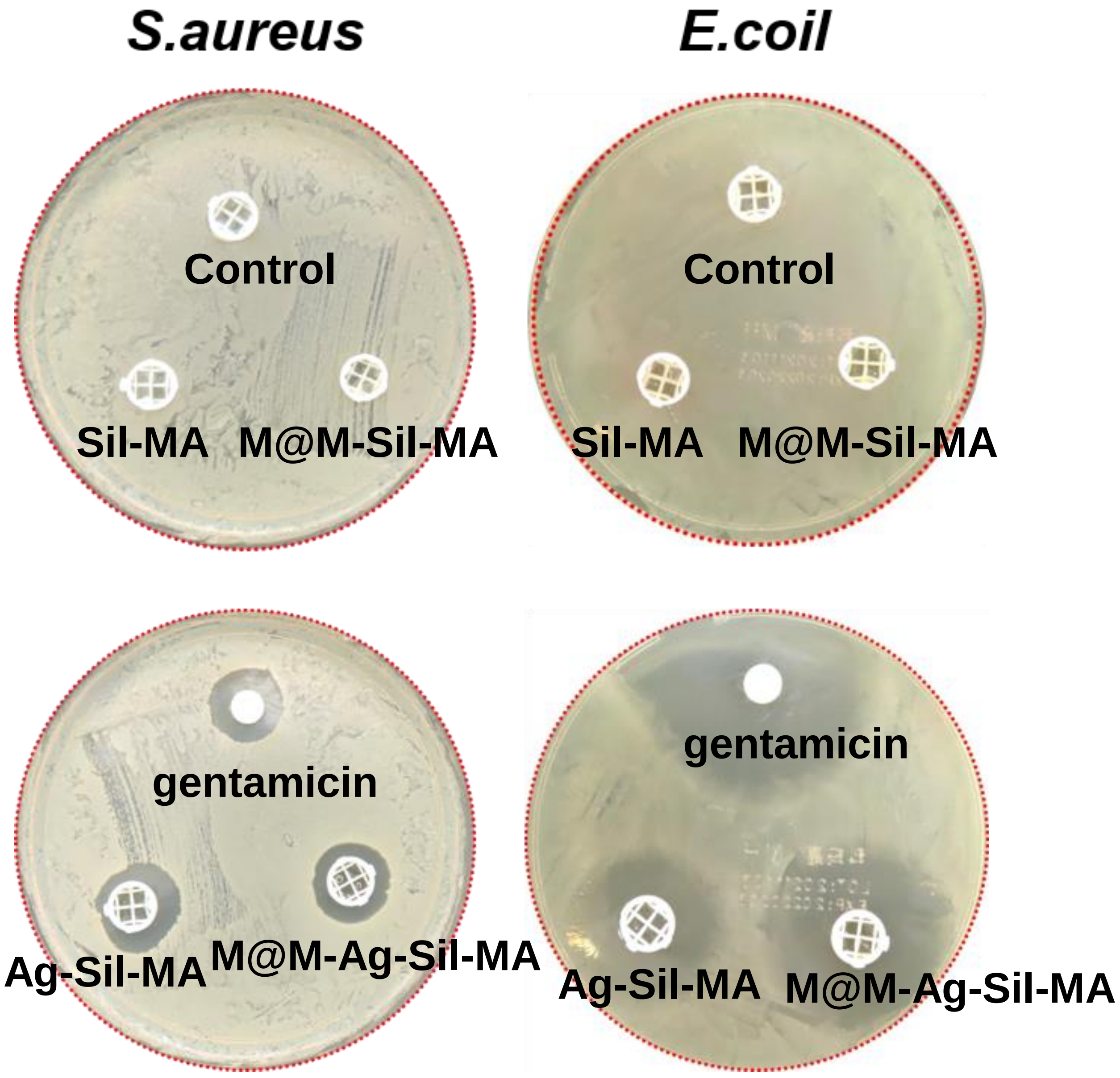

Fig S12. Representative photos of the hydrogel systems and gentamicin disks of ZOI against *S. aureus* and *E. coli* at day 7.

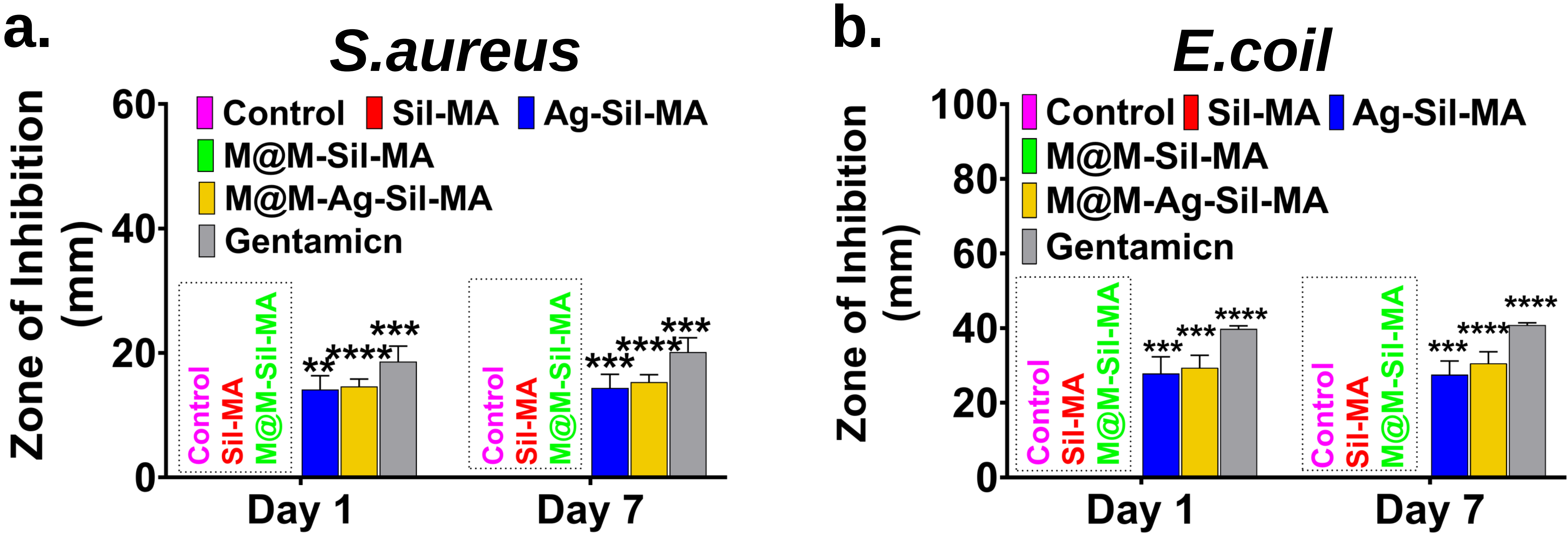

Fig S13.Quantitative data of the hydrogel systems and gentamicin disks of ZOI against a). *S. aureus* and b). *E. coli*.

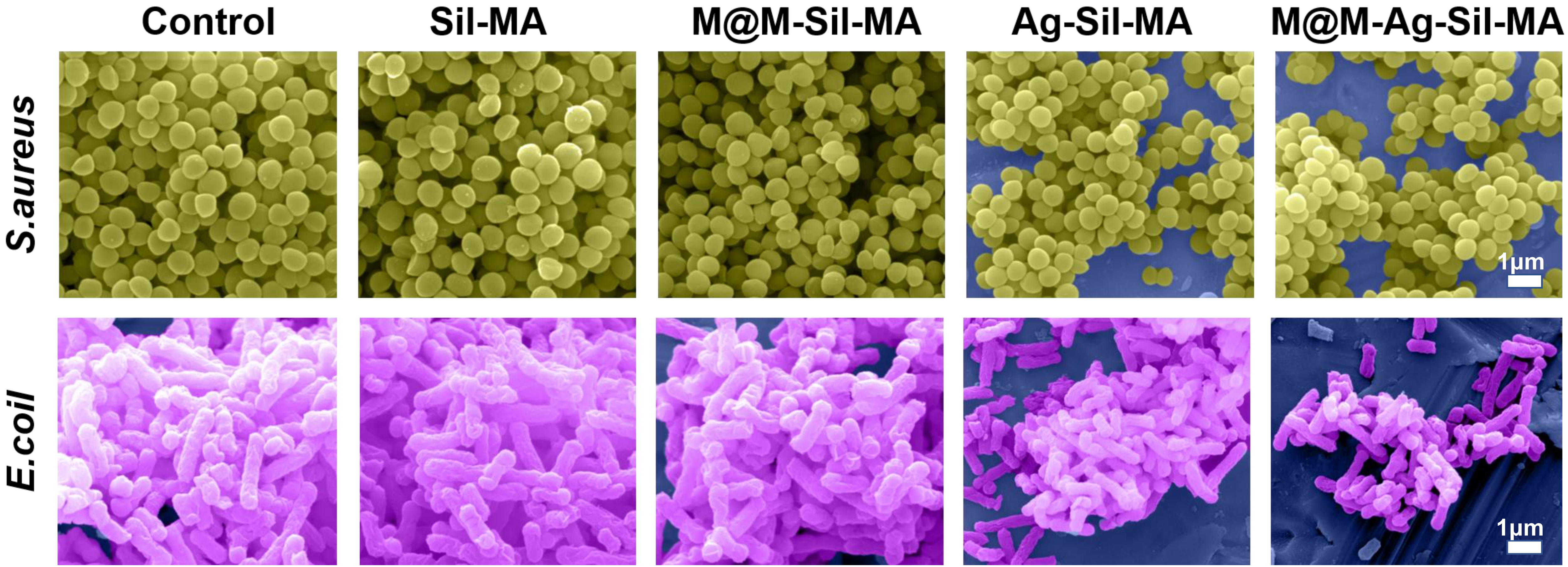

Fig S14. Representative SEM images of bacteria. Yellow spheres indicate *S.aureus*, and violet rods indicate *E. coli*.

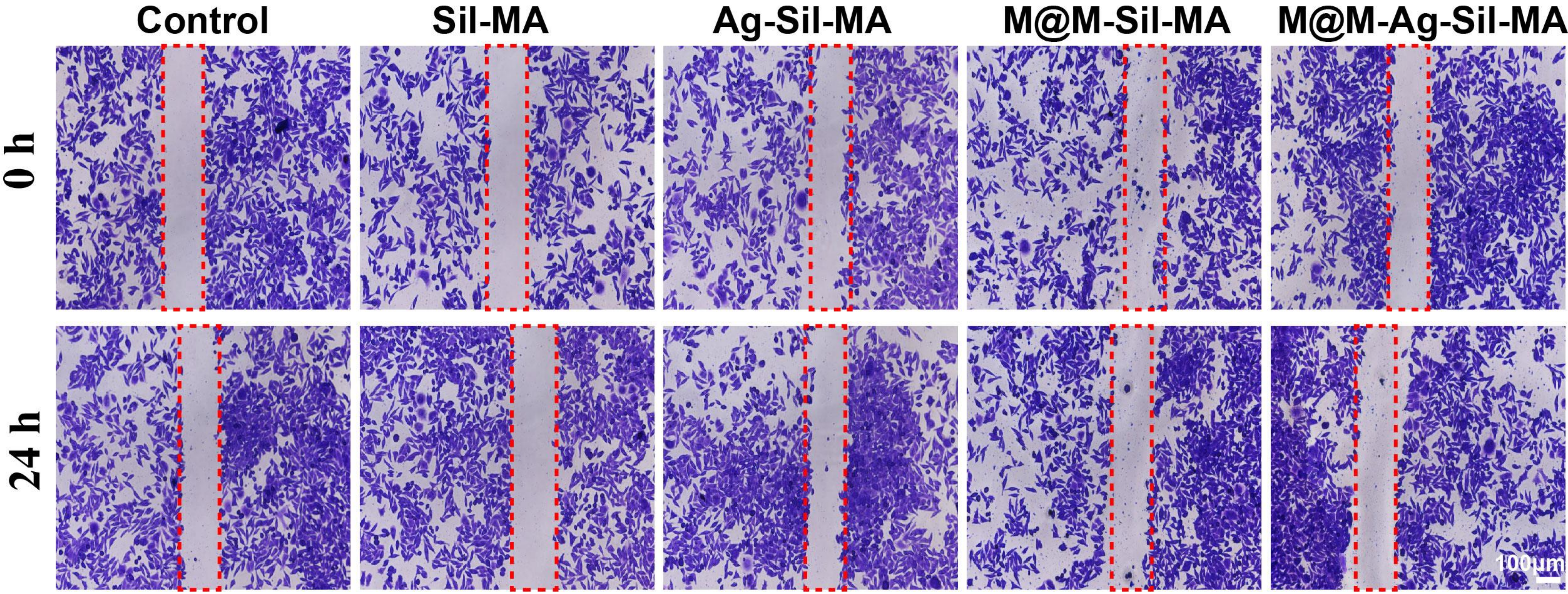

Fig S15. Pictures of migration of L929 cells treated directly by CM on days 7.(Scale bar: 100μm).

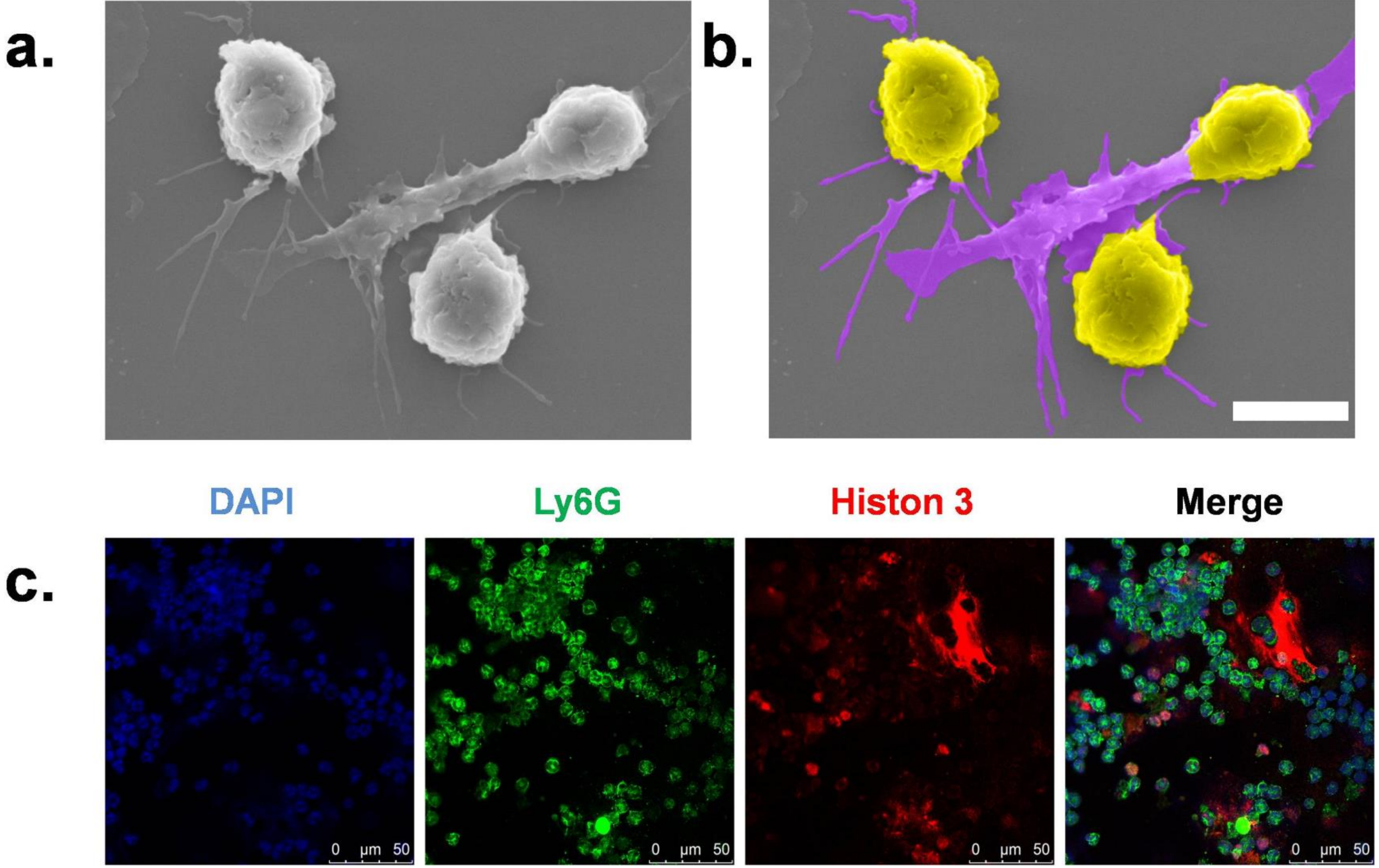

Fig S16. SEM and Fluorescence images of NETs formation. a). The raw SEM images of NETs formation treated with LPS b). The processed images of NETs. Yellow area indicated neutrophils. Purple area indicated NETs.(Scale Bar: 5µm) c). Fluorescence images of NETs formation. DAPI indicated nucleus; Ly6G is the surface marker of neutrophils. Histon 3 is the positive marker of NETs.(Scale Bar: 50µm)

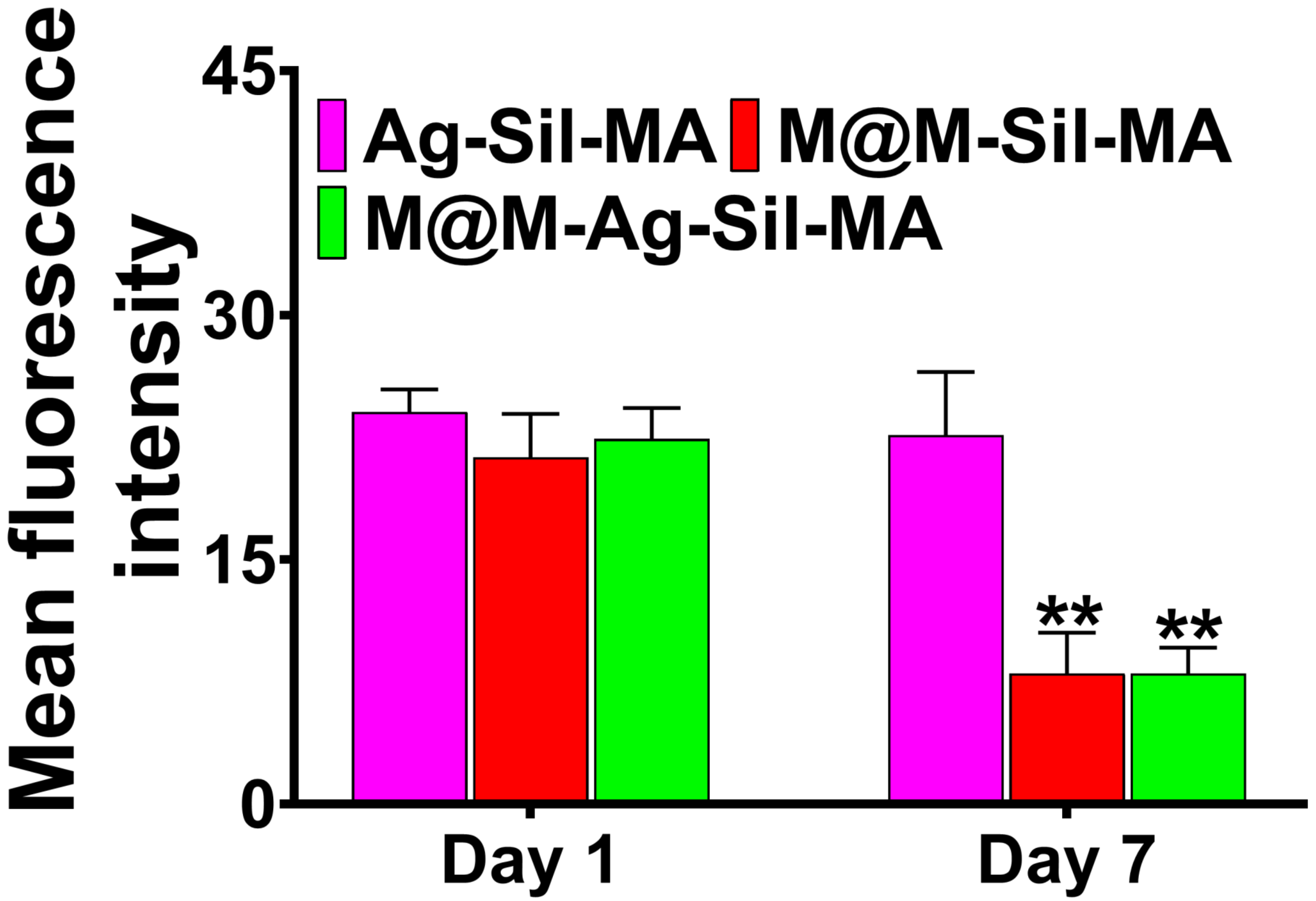

Fig S17. Fluorescence intensity of NETs of LPS-treated neutrophils

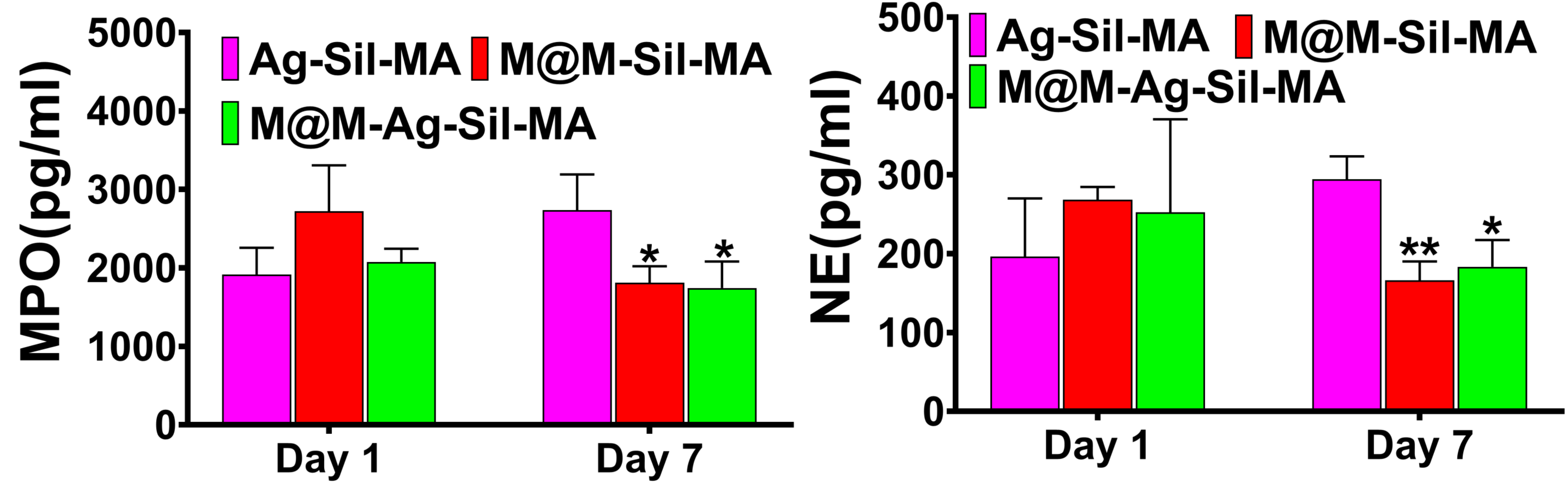

Fig S18. ELISA results for MPO and NE of LPS-treated neutrophils.

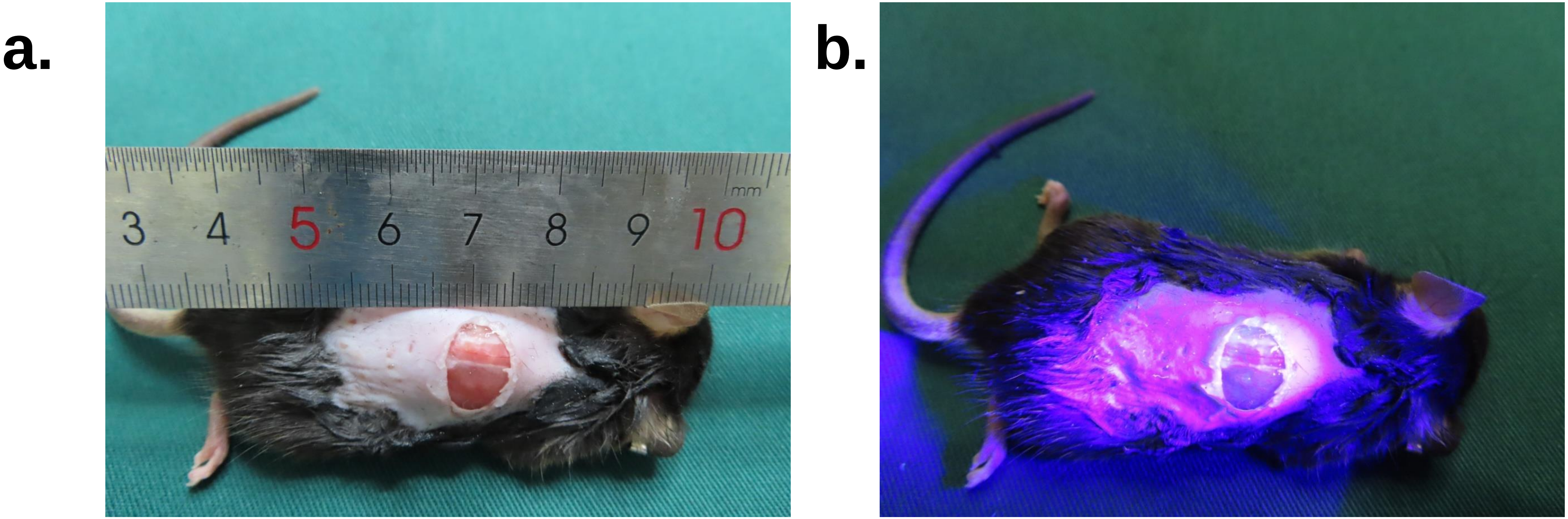

Fig S19. The digital images of diabetic wound model of mice. a).The image of wound model created by a punch. b). The image of hydrogel photocured *in situ.*

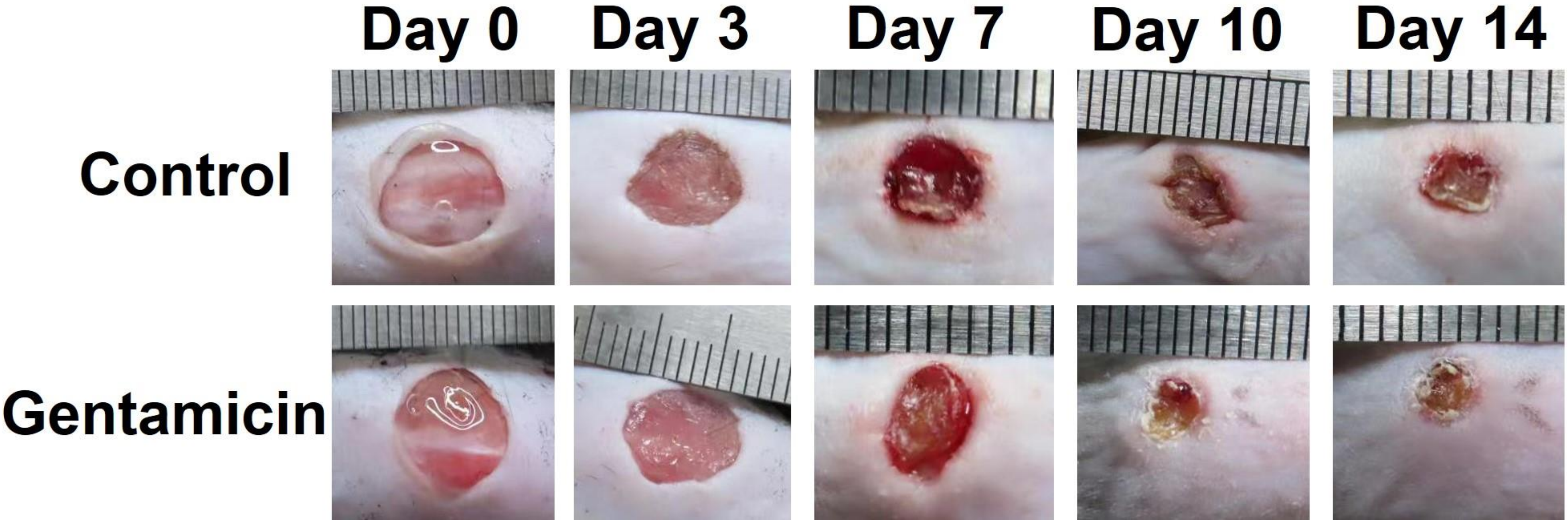

Fig S20. Photographs of the wounds with different treatments （Control Gentamicin） on days 0, 3, 7, 10 and 14.

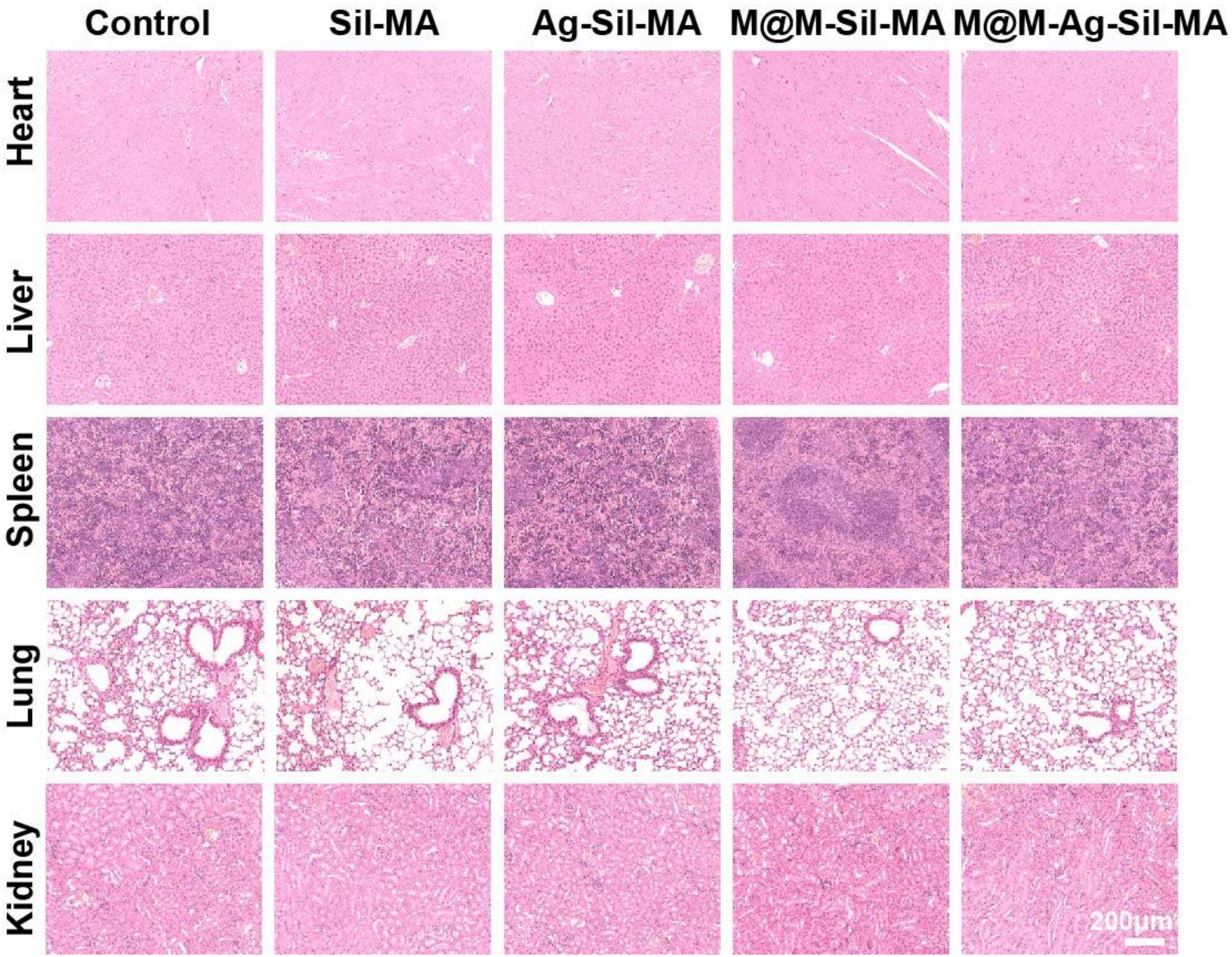

Fig S21. HE staining of pathological sections from major organs in different group （Scale bar： 200μm）.

Gene	Upper Primer Sequence	Lower Primer Sequence
Gapdh	5' AAA TGG TGA AGG TCG GTG TG 3'	5' AGG TCA ATG AAG GGG TCG TT 3'
iNOS	5' TTG ACG CTC GGA ACT GTA 3'	5' GTT GGT GGC ATA AAG TAT GTG 3'
Arg-1	5' GCC AGG GAC TGA CTA CCT TAA 3'	5' AGT TCT GTC TGC TTT GCT GTG 3'

Table S1. Primers used for RT-PCR.
